# Supplementary material for: Metacommunity analyses show an increase in ecological specialisation throughout the Ediacaran period
Source: PLoS Biol. 2022 May 17;20(5):e3001289. doi: 10.1371/journal.pbio.3001289 (PMC9113585; doi:10.1371/journal.pbio.3001289)
Supplement: S1 Table — (DOCX) [file pbio.3001289.s005.docx]

|  |  | **Metrics** | | |
| --- | --- | --- | --- | --- |
| **Community type** | **Properties** | **Coherence** | **Range Turnover** | **Boundary Clumping** |
| **Random** | No structure | Random |  |  |
| **Checkerboard** | High number mutually exclusive pairs; taxa do not respond to gradient | Negative |  |  |
| **Nested clumped** | Species poor communities subsets species rich communities; community synchronous response to gradient | Positive | Negative | Positive |
| **Nested random** | Species poor communities are random subsets species rich communities | Positive | Negative | Not significant |
| **Nested hyperdispersed** | Species poor communities subsets species rich communities; species respond individualistically to gradient | Positive | Negative | Negative |
| **Clemenstsian** | Community synchronous response to gradient | Positive | Positive | Positive |
| **Gleasonian** | Species respond individualistically to gradient | Positive | Positive | Not significant |
| **Evenly spaced** | Species spread along gradient with little overlap | Positive | Positive | Negative |
| **Quasi-nested clumped** | Species poor communities subsets species rich communities; community synchronous response to gradient, fewer turnover than random but not significant | Positive | Negative but not significant | Positive |
| **Quasi-nested hyperdispersed** | Species poor communities are random subsets species rich communities; fewer replacements than random but not significant | Positive | Negative but not significant | Not significant |
| **Quasi - nested random** | Species poor communities subsets species rich communities; species respond individualistically to gradient; fewer replacement than random but not significant | Positive | Negative but not significant | Negative |
| **Quasi-Clementsian** | Community synchronous response to gradient; more replacements than random but not significant | Positive | Positive but not significant | Positive |
| **Quasi- Gleasonian** | Species respond individualistically to gradient more replacements than random but not significant | Positive | Positive but not significant | Not significant |
| **Quasi-evenly spaced** | Species spread along gradient with little overlap; more replacements than random but not significant | Positive | Positive but not significant | Negative |

Table S1: Summary table of how metacommunity properties are expressed in terms of the EMS metrics
