# Supplementary material for: Metacommunity analyses show an increase in ecological specialisation throughout the Ediacaran period
Source: PLoS Biol. 2022 May 17;20(5):e3001289. doi: 10.1371/journal.pbio.3001289 (PMC9113585; doi:10.1371/journal.pbio.3001289)
Supplement: S2 Table — Sp1_inc is the number of sites that have taxa 1. Obc_cooccur is the observed number of sites with both species. Prob_cooccur is the probability both species occur at a site. Exp_cooccur is the expected number of sites having both taxa. P_Lt is the probability that the 2 taxa would co-occur at a frequency less than observed, and P_gt is the probability that the 2 taxa would co-occur at a frequency greater than observed. Difference is the difference between observed and expected probabilities, where difference > 0.95 the association is considered significant. (DOCX) [file pbio.3001289.s006.docx]

| **Species 1** | **Species 2** | **sp1**  **_inc** | **sp2**  **_inc** | **obs**  **_cooccur** | **prob**  **_cooccur** | **exp**  **_cooccur** | **p_lt** | **p_gt** | **Species 1 Name** | **Species 2 Name** | **Association** | **Difference** |
| --- | --- | --- | --- | --- | --- | --- | --- | --- | --- | --- | --- | --- |
| 15 | 59 | 26 | 12 | 11 | 0.042 | 3.600 | 1.000 | 0.000 | *Charnia* | *Primocandelabrum* | Positive | 1.000 |
| 24 | 41 | 18 | 10 | 9 | 0.024 | 2.100 | 1.000 | 0.000 | *Dickinsonia* | *Kimberella* | Positive | 1.000 |
| 24 | 57 | 18 | 12 | 11 | 0.029 | 2.500 | 1.000 | 0.000 | *Dickinsonia* | *Parvancorina* | Positive | 1.000 |
| 24 | 77 | 18 | 13 | 13 | 0.032 | 2.700 | 1.000 | 0.000 | *Dickinsonia* | *Tribrachidium* | Positive | 1.000 |
| 41 | 57 | 10 | 12 | 8 | 0.016 | 1.400 | 1.000 | 0.000 | *Kimberella* | *Parvancorina* | Positive | 1.000 |
| 41 | 77 | 10 | 13 | 8 | 0.018 | 1.500 | 1.000 | 0.000 | *Kimberella* | *Tribrachidium* | Positive | 1.000 |
| 57 | 77 | 12 | 13 | 11 | 0.021 | 1.800 | 1.000 | 0.000 | *Parvancorina* | *Tribrachidium* | Positive | 1.000 |
| 24 | 74 | 18 | 6 | 6 | 0.015 | 1.300 | 1.000 | 0.000 | *Dickinsonia* | *Temnoxa* | Positive | 1.000 |
| 14 | 30 | 16 | 10 | 7 | 0.022 | 1.900 | 1.000 | 0.000 | *Bradgatia* | *Fractofusus* | Positive | 1.000 |
| 2 | 24 | 5 | 18 | 5 | 0.012 | 1.000 | 1.000 | 0.000 | *Andiva* | *Dickinsonia* | Positive | 1.000 |
| 23 | 24 | 5 | 18 | 5 | 0.012 | 1.000 | 1.000 | 0.000 | *Cyanorus* | *Dickinsonia* | Positive | 1.000 |
| 24 | 63 | 18 | 5 | 5 | 0.012 | 1.000 | 1.000 | 0.000 | *Dickinsonia* | *Rugoconites* | Positive | 1.000 |
| 24 | 82 | 18 | 5 | 5 | 0.012 | 1.000 | 1.000 | 0.000 | *Dickinsonia* | *Yorgia* | Positive | 1.000 |
| 15 | 16 | 26 | 35 | 18 | 0.123 | 10.60 | 1.000 | 0.000 | *Charnia* | *Charniodiscus* | Positive | 0.999 |
| 61 | 62 | 11 | 8 | 5 | 0.012 | 1.000 | 1.000 | 0.001 | *Pteridinium* | *Rangea* | Positive | 0.999 |
| 14 | 59 | 16 | 12 | 7 | 0.026 | 2.200 | 1.000 | 0.001 | *Bradgatia* | *Primocandelabrum* | Positive | 0.999 |
| 24 | 52 | 18 | 6 | 5 | 0.015 | 1.300 | 1.000 | 0.001 | *Dickinsonia* | *Onega* | Positive | 0.999 |
| 15 | 35 | 26 | 5 | 5 | 0.018 | 1.500 | 1.000 | 0.002 | *Charnia* | *Hadrynichorde* | Positive | 0.998 |
| 14 | 16 | 16 | 35 | 12 | 0.076 | 6.500 | 1.000 | 0.003 | *Bradgatia* | *Charniodiscus* | Positive | 0.997 |
| 14 | 15 | 16 | 26 | 10 | 0.056 | 4.800 | 1.000 | 0.003 | *Bradgatia* | *Charnia* | Positive | 0.996 |
| 12 | 14 | 8 | 16 | 5 | 0.017 | 1.500 | 1.000 | 0.005 | *Beothukis* | *Bradgatia* | Positive | 0.995 |
| 12 | 15 | 8 | 26 | 6 | 0.028 | 2.400 | 0.999 | 0.008 | *Beothukis* | *Charnia* | Positive | 0.991 |
| 24 | 62 | 18 | 8 | 5 | 0.019 | 1.700 | 0.999 | 0.009 | *Dickinsonia* | *Rangea* | Positive | 0.990 |
| 16 | 30 | 35 | 10 | 8 | 0.047 | 4.100 | 0.999 | 0.010 | *Charniodiscus* | *Fractofusus* | Positive | 0.989 |
| 57 | 62 | 12 | 8 | 4 | 0.013 | 1.100 | 0.999 | 0.012 | *Parvancorina* | *Rangea* | Positive | 0.987 |
| 16 | 59 | 35 | 12 | 9 | 0.057 | 4.900 | 0.998 | 0.011 | *Charniodiscus* | *Primocandelabrum* | Positive | 0.987 |
| 62 | 77 | 8 | 13 | 4 | 0.014 | 1.200 | 0.998 | 0.016 | *Rangea* | *Tribrachidium* | Positive | 0.982 |
| 51 | 77 | 9 | 13 | 4 | 0.016 | 1.400 | 0.997 | 0.027 | *Nimbia* | *Tribrachidium* | Positive | 0.970 |
| 24 | 54 | 18 | 10 | 5 | 0.024 | 2.100 | 0.995 | 0.030 | *Dickinsonia* | *Palaeopascichnus* | Positive | 0.965 |
| 15 | 30 | 26 | 10 | 6 | 0.035 | 3.000 | 0.993 | 0.039 | *Charnia* | *Fractofusus* | Positive | 0.954 |
| 54 | 77 | 10 | 13 | 4 | 0.018 | 1.500 | 0.994 | 0.040 | *Palaeopascichnus* | *Tribrachidium* | Positive | 0.954 |
| 16 | 17 | 35 | 12 | 0 | 0.057 | 4.900 | 0.001 | 1.000 | *Charniodiscus* | *Cloudina* | Negative | 0.999 |
| 15 | 17 | 26 | 12 | 0 | 0.042 | 3.600 | 0.009 | 1.000 | *Charnia* | *Cloudina* | Negative | 0.991 |
| 14 | 24 | 16 | 18 | 0 | 0.039 | 3.300 | 0.015 | 1.000 | *Bradgatia* | *Dickinsonia* | Negative | 0.985 |
| 16 | 28 | 35 | 7 | 0 | 0.033 | 2.800 | 0.022 | 1.000 | *Charniodiscus* | *Ernietta* | Negative | 0.978 |
| 11 | 14 | 14 | 16 | 0 | 0.030 | 2.600 | 0.043 | 1.000 | *Beltanelliformis* | *Bradgatia* | Negative | 0.957 |
| 17 | 24 | 12 | 18 | 0 | 0.029 | 2.500 | 0.048 | 1.000 | *Cloudina* | *Dickinsonia* | Negative | 0.952 |
| 24 | 59 | 18 | 12 | 0 | 0.029 | 2.500 | 0.048 | 1.000 | *Dickinsonia* | *Primocandelabrum* | Negative | 0.952 |

Table S2: Co-occurrence analysis for the total dataset showing only significant associations

Sp1_inc is the number of sites which have taxa 1. Obc_cooccur is the observed number of sites with both species. Prob_cooccur is the probability both species occur at a site. Exp_cooccur is the expected number of sites having both taxa. P_Lt probably that the two taxa would co-occur at a frequency less than observed and P_gt is the probability that the two taxa would co-occur at a frequency greater than observed. Difference is the difference between observed and expected probabilities. Where difference > 0.95 the association is considered significant.
