# Supplementary material for: Metacommunity analyses show an increase in ecological specialisation throughout the Ediacaran period
Source: PLoS Biol. 2022 May 17;20(5):e3001289. doi: 10.1371/journal.pbio.3001289 (PMC9113585; doi:10.1371/journal.pbio.3001289)
Supplement: S3 Table — Sp1_inc is the number of sites that have taxa 1. Obc_cooccur is the observed number of sites with both species. Prob_cooccur is the probability both species occur at a site. Exp_cooccur is the expected number of sites having both taxa. P_Lt is the probability that the 2 taxa would co-occur at a frequency less than observed, and P_gt is the probability that the 2 taxa would co-occur at a frequency greater than observed. Difference is the difference between observed and expected probabilities, where difference > 0.95 the association is considered significant. (DOCX) [file pbio.3001289.s007.docx]

| **Species 1** | **Species 2** | **sp1**  **_inc** | **sp2**  **_inc** | **obs**  **_cooccur** | **prob**  **_cooccur** | **exp**  **_cooccur** | **p_lt** | **p_gt** | **Species 1 Name** | **Species 2 Name** | **Association** | **Difference** |
| --- | --- | --- | --- | --- | --- | --- | --- | --- | --- | --- | --- | --- |
| 2 | 7 | 7 | 9 | 5 | 0.070 | 2.100 | 0.999 | 0.014 | *Beothukis* | *Fractofusus* | Positive | 0.985 |
| 4 | 12 | 19 | 11 | 10 | 0.232 | 7.000 | 0.999 | 0.020 | *Charnia* | *Primocandelabrum* | Positive | 0.979 |
| 3 | 5 | 15 | 18 | 12 | 0.300 | 9.000 | 0.996 | 0.030 | *Bradgatia* | *Charniodiscus* | Positive | 0.966 |
| 4 | 11 | 19 | 5 | 1 | 0.106 | 3.200 | 0.047 | 0.997 | *Charnia* | *Pectinifrons* | Negative | 0.950 |
| 9 | 12 | 5 | 11 | 4 | 0.061 | 1.800 | 0.997 | 0.047 | *Hadrynichorde* | *Primocandelabrum* | Positive | 0.950 |

Table S3: Co-occurrence analysis for the Avalonian dataset showing only significant associations.

Sp1_inc is the number of sites which have taxa 1. Obc_cooccur is the observed number of sites with both species. Prob_cooccur is the probability both species occur at a site. Exp_cooccur is the expected number of sites having both taxa. P_Lt probably that the two taxa would co-occur at a frequency less than observed and P_gt is the probability that the two taxa would co-occur at a frequency greater than observed. Difference is the difference between observed and expected probabilities. Where difference > 0.95 the association is considered significant.
