# Supplementary material for: Metacommunity analyses show an increase in ecological specialisation throughout the Ediacaran period
Source: PLoS Biol. 2022 May 17;20(5):e3001289. doi: 10.1371/journal.pbio.3001289 (PMC9113585; doi:10.1371/journal.pbio.3001289)
Supplement: S5 Table — Sp1_inc is the number of sites that have taxa 1. Obc_cooccur is the observed number of sites with both species. Prob_cooccur is the probability both species occur at a site. Exp_cooccur is the expected number of sites having both taxa. P_Lt is the probability that the 2 taxa would co-occur at a frequency less than observed, and P_gt is the probability that the 2 taxa would co-occur at a frequency greater than observed. Difference is the difference between observed and expected probabilities, where difference > 0.95 the association is considered significant. (DOCX) [file pbio.3001289.s009.docx]

| **Species 1** | **Species 2** | **sp1**  **_inc** | **sp2**  **_inc** | **obs**  **_cooccur** | **prob**  **_cooccur** | **exp**  **_cooccur** | **p_lt** | **p_gt** | **Species 1 Name** | **Species 2 Name** | **Association** | **Difference** |
| --- | --- | --- | --- | --- | --- | --- | --- | --- | --- | --- | --- | --- |
| 2 | 8 | 7 | 8 | 5 | 0.106 | 2.400 | 0.998 | 0.026 | *Beothukis* | *Fractofusus* | Positive | 0.973 |
| 3 | 5 | 13 | 13 | 10 | 0.319 | 7.300 | 0.997 | 0.033 | *Bradgatia* | *Charniodiscus* | Positive | 0.964 |

Table S5: Co-occurrence analysis for the Avalonian margin slope dataset showing only significant associations.

Sp1_inc is the number of sites which have taxa 1. Obc_cooccur is the observed number of sites with both species. Prob_cooccur is the probability both species occur at a site. Exp_cooccur is the expected number of sites having both taxa. P_Lt probably that the two taxa would co-occur at a frequency less than observed and P_gt is the probability that the two taxa would co-occur at a frequency greater than observed. Difference is the difference between observed and expected probabilities. Where difference > 0.95 the association is considered significant.
