# Supplementary material for: Metacommunity analyses show an increase in ecological specialisation throughout the Ediacaran period
Source: PLoS Biol. 2022 May 17;20(5):e3001289. doi: 10.1371/journal.pbio.3001289 (PMC9113585; doi:10.1371/journal.pbio.3001289)
Supplement: S6 Table — Sp1_inc is the number of sites that have taxa 1. Obc_cooccur is the observed number of sites with both species. Prob_cooccur is the probability both species occur at a site. Exp_cooccur is the expected number of sites having both taxa. P_Lt is the probability that the 2 taxa would co-occur at a frequency less than observed, and P_gt is the probability that the 2 taxa would co-occur at a frequency greater than observed. Difference is the difference between observed and expected probabilities, where difference > 0.95 the association is considered significant. (DOCX) [file pbio.3001289.s010.docx]

| **sp1_inc** | **sp2_inc** | **obs_cooccur** | **prob_cooccur** | **exp_cooccur** | **p_lt** | **p_gt** | **Species 1 Name** | **Species 2 Name** | **Association** | **p-value** |
| --- | --- | --- | --- | --- | --- | --- | --- | --- | --- | --- |
| 12 | 13 | 11 | 0.185 | 5.400 | 1.000 | 0.000 | *Parvancorina* | *Tribrachidium* | Positive | 1.000 |
| 17 | 13 | 13 | 0.263 | 7.600 | 1.000 | 0.000 | *Dickinsonia* | *Tribrachidium* | Positive | 1.000 |
| 9 | 12 | 8 | 0.128 | 3.700 | 1.000 | 0.001 | *Kimberella* | *Parvancorina* | Positive | 0.999 |
| 5 | 9 | 5 | 0.054 | 1.600 | 1.000 | 0.001 | *Andiva* | *Kimberella* | Positive | 0.999 |
| 5 | 9 | 5 | 0.054 | 1.600 | 1.000 | 0.001 | *Cyanorus* | *Kimberella* | Positive | 0.999 |
| 6 | 12 | 6 | 0.086 | 2.500 | 1.000 | 0.002 | *Onega* | *Parvancorina* | Positive | 0.998 |
| 9 | 13 | 8 | 0.139 | 4.000 | 1.000 | 0.002 | *Kimberella* | *Tribrachidium* | Positive | 0.998 |
| 17 | 9 | 9 | 0.182 | 5.300 | 1.000 | 0.002 | *Dickinsonia* | *Kimberella* | Positive | 0.998 |
| 5 | 6 | 4 | 0.036 | 1.000 | 1.000 | 0.003 | *Andiva* | *Onega* | Positive | 0.997 |
| 17 | 12 | 11 | 0.243 | 7.000 | 1.000 | 0.003 | *Dickinsonia* | *Parvancorina* | Positive | 0.997 |
| 9 | 6 | 5 | 0.064 | 1.900 | 1.000 | 0.005 | *Kimberella* | *Onega* | Positive | 0.994 |
| 9 | 6 | 5 | 0.064 | 1.900 | 1.000 | 0.005 | *Kimberella* | *Temnoxa* | Positive | 0.994 |
| 5 | 12 | 5 | 0.071 | 2.100 | 1.000 | 0.007 | *Andiva* | *Parvancorina* | Positive | 0.993 |
| 12 | 5 | 5 | 0.071 | 2.100 | 1.000 | 0.007 | *Charniodiscus* | *Rangea* | Positive | 0.993 |
| 6 | 6 | 4 | 0.043 | 1.200 | 1.000 | 0.008 | *Onega* | *Temnoxa* | Positive | 0.991 |
| 10 | 4 | 4 | 0.048 | 1.400 | 1.000 | 0.009 | *Beltanelliformis* | *Nemiana* | Positive | 0.991 |
| 5 | 13 | 5 | 0.077 | 2.200 | 1.000 | 0.011 | *Andiva* | *Tribrachidium* | Positive | 0.989 |
| 5 | 13 | 5 | 0.077 | 2.200 | 1.000 | 0.011 | *Rugoconites* | *Tribrachidium* | Positive | 0.989 |
| 6 | 7 | 4 | 0.050 | 1.400 | 0.999 | 0.018 | *Charnia* | *Nimbia* | Positive | 0.981 |
| 4 | 12 | 4 | 0.057 | 1.700 | 1.000 | 0.021 | *Albumares* | *Parvancorina* | Positive | 0.979 |
| 4 | 12 | 4 | 0.057 | 1.700 | 1.000 | 0.021 | *Armillifera* | *Parvancorina* | Positive | 0.979 |
| 9 | 5 | 4 | 0.054 | 1.600 | 0.999 | 0.022 | *Kimberella* | *Yorgia* | Positive | 0.977 |
| 17 | 6 | 6 | 0.121 | 3.500 | 1.000 | 0.026 | *Dickinsonia* | *Temnoxa* | Positive | 0.974 |
| 4 | 13 | 4 | 0.062 | 1.800 | 1.000 | 0.030 | *Albumares* | *Tribrachidium* | Positive | 0.970 |
| 4 | 13 | 4 | 0.062 | 1.800 | 1.000 | 0.030 | *Armillifera* | *Tribrachidium* | Positive | 0.970 |
| 6 | 12 | 5 | 0.086 | 2.500 | 0.998 | 0.030 | *Charnia* | *Parvancorina* | Positive | 0.968 |
| 12 | 6 | 5 | 0.086 | 2.500 | 0.998 | 0.030 | *Parvancorina* | *Temnoxa* | Positive | 0.968 |
| 6 | 13 | 5 | 0.093 | 2.700 | 0.996 | 0.047 | *Temnoxa* | *Tribrachidium* | Positive | 0.953 |
| 6 | 13 | 5 | 0.093 | 2.700 | 0.996 | 0.047 | *Onega* | *Tribrachidium* | Positive | 0.953 |
| 6 | 13 | 5 | 0.093 | 2.700 | 0.996 | 0.047 | *Charnia* | *Tribrachidium* | Positive | 0.953 |
| 5 | 6 | 3 | 0.036 | 1.000 | 0.997 | 0.046 | *Andiva* | *Temnoxa* | Positive | 0.951 |
| 6 | 5 | 3 | 0.036 | 1.000 | 0.997 | 0.046 | *Charnia* | *Rangea* | Positive | 0.951 |
| 5 | 6 | 3 | 0.036 | 1.000 | 0.997 | 0.046 | *Cyanorus* | *Onega* | Positive | 0.951 |
| 5 | 6 | 3 | 0.036 | 1.000 | 0.997 | 0.046 | *Cyanorus* | *Temnoxa* | Positive | 0.951 |
| 6 | 5 | 3 | 0.036 | 1.000 | 0.997 | 0.046 | *Onega* | *Yorgia* | Positive | 0.951 |

Table S6. Co-occurrence analysis for the White Sea dataset showing only significant associations.

Sp1_inc is the number of sites which have taxa 1. Obc_cooccur is the observed number of sites with both species. Prob_cooccur is the probability both species occur at a site. Exp_cooccur is the expected number of sites having both taxa. P_Lt probably that the two taxa would co-occur at a frequency less than observed and P_gt is the probability that the two taxa would co-occur at a frequency greater than observed. Difference is the difference between observed and expected probabilities. Where difference > 0.95 the association is considered significant.
