# Supplementary material for: Metacommunity analyses show an increase in ecological specialisation throughout the Ediacaran period
Source: PLoS Biol. 2022 May 17;20(5):e3001289. doi: 10.1371/journal.pbio.3001289 (PMC9113585; doi:10.1371/journal.pbio.3001289)
Supplement: S8 Table — Sp1_inc is the number of sites that have taxa 1. Obc_cooccur is the observed number of sites with both species. Prob_cooccur is the probability both species occur at a site. Exp_cooccur is the expected number of sites having both taxa. P_Lt is the probability that the 2 taxa would co-occur at a frequency less than observed, and P_gt is the probability that the 2 taxa would co-occur at a frequency greater than observed. Difference is the difference between observed and expected probabilities, where difference > 0.95 the association is considered significant. (DOCX) [file pbio.3001289.s012.docx]

| **Species 1** | **Species 2** | **sp1**  **_inc** | **sp2**  **_inc** | **obs**  **_cooccur** | **prob**  **_cooccur** | **exp**  **_cooccur** | **p_lt** | **p_gt** | **Species 1 Name** | **Species 2 Name** | **Association** | **Difference** |
| --- | --- | --- | --- | --- | --- | --- | --- | --- | --- | --- | --- | --- |
| 2 | 48 | 3 | 3 | 3 | 0.111 | 1.00 | 1.000 | 0.012 | *Andiva* | *Yorgia* | Positive | 0.988 |
| 16 | 43 | 6 | 6 | 6 | 0.444 | 4.00 | 1.000 | 0.012 | *Dickinsonia* | *Tribrachidium* | Positive | 0.988 |
| 20 | 29 | 4 | 5 | 4 | 0.247 | 2.20 | 1.000 | 0.040 | *Kimberella* | *Parvancorina* | Positive | 0.960 |
| 2 | 20 | 3 | 4 | 3 | 0.148 | 1.30 | 1.000 | 0.048 | *Andiva* | *Kimberella* | Positive | 0.952 |
| 16 | 29 | 6 | 5 | 5 | 0.370 | 3.30 | 1.000 | 0.048 | *Dickinsonia* | *Parvancorina* | Positive | 0.952 |
| 20 | 48 | 4 | 3 | 3 | 0.148 | 1.30 | 1.000 | 0.048 | *Kimberella* | *Yorgia* | Positive | 0.952 |
| 29 | 43 | 5 | 6 | 5 | 0.370 | 3.30 | 1.000 | 0.048 | *Parvancorina* | *Tribrachidium* | Positive | 0.952 |
| 8 | 20 | 4 | 4 | 0 | 0.198 | 1.80 | 0.040 | 1.000 | *Beltanelliformis* | *Kimberella* | Negative | 0.960 |

Table S8: Co-occurrence analysis for the White Sea deep subtidal dataset showing only significant associations.

Sp1_inc is the number of sites which have taxa 1. Obc_cooccur is the observed number of sites with both species. Prob_cooccur is the probability both species occur at a site. Exp_cooccur is the expected number of sites having both taxa. P_Lt probably that the two taxa would co-occur at a frequency less than observed and P_gt is the probability that the two taxa would co-occur at a frequency greater than observed. Difference is the difference between observed and expected probabilities. Where difference > 0.95 the association is considered significant.
