# Supplementary material for: Metacommunity analyses show an increase in ecological specialisation throughout the Ediacaran period
Source: PLoS Biol. 2022 May 17;20(5):e3001289. doi: 10.1371/journal.pbio.3001289 (PMC9113585; doi:10.1371/journal.pbio.3001289)
Supplement: S9 Table — (DOCX) [file pbio.3001289.s013.docx]

| **Species 1** | **Species 2** | **sp1**  **_inc** | **sp2**  **_inc** | **obs**  **_cooccur** | **prob**  **_cooccur** | **exp**  **_cooccur** | **p_lt** | **p_gt** | **Species 1 Name** | **Species 2 Name** | **Association** | **Difference** |
| --- | --- | --- | --- | --- | --- | --- | --- | --- | --- | --- | --- | --- |
| 23 | 27 | 6 | 8 | 6 | 0.133 | 2.500 | 1.000 | 0.001 | *Onega* | *Parvancorina* | Positive | 0.999 |
| 27 | 35 | 8 | 8 | 7 | 0.177 | 3.400 | 1.000 | 0.001 | *Parvancorina* | *Tribrachidium* | Positive | 0.999 |
| 7 | 22 | 4 | 5 | 4 | 0.055 | 1.100 | 1.000 | 0.001 | *Charnia* | *Nimbia* | Positive | 0.999 |
| 1 | 17 | 5 | 7 | 5 | 0.097 | 1.800 | 1.000 | 0.002 | *Andiva* | *Kimberella* | Positive | 0.998 |
| 10 | 17 | 5 | 7 | 5 | 0.097 | 1.800 | 1.000 | 0.002 | *Cyanorus* | *Kimberella* | Positive | 0.998 |
| 11 | 35 | 11 | 8 | 8 | 0.244 | 4.600 | 1.000 | 0.002 | *Dickinsonia* | *Tribrachidium* | Positive | 0.998 |
| 1 | 27 | 5 | 8 | 5 | 0.111 | 2.100 | 1.000 | 0.005 | *Andiva* | *Parvancorina* | Positive | 0.995 |
| 1 | 35 | 5 | 8 | 5 | 0.111 | 2.100 | 1.000 | 0.005 | *Andiva* | *Tribrachidium* | Positive | 0.995 |
| 1 | 37 | 5 | 5 | 4 | 0.069 | 1.300 | 1.000 | 0.006 | *Andiva* | *Yorgia* | Positive | 0.994 |
| 17 | 27 | 7 | 8 | 6 | 0.155 | 2.900 | 1.000 | 0.006 | *Kimberella* | *Parvancorina* | Positive | 0.994 |
| 17 | 35 | 7 | 8 | 6 | 0.155 | 2.900 | 1.000 | 0.006 | *Kimberella* | *Tribrachidium* | Positive | 0.994 |
| 11 | 17 | 11 | 7 | 7 | 0.213 | 4.100 | 1.000 | 0.007 | *Dickinsonia* | *Kimberella* | Positive | 0.993 |
| 17 | 23 | 7 | 6 | 5 | 0.116 | 2.200 | 1.000 | 0.010 | *Kimberella* | *Onega* | Positive | 0.990 |
| 17 | 34 | 7 | 6 | 5 | 0.116 | 2.200 | 1.000 | 0.010 | *Kimberella* | *Temnoxa* | Positive | 0.990 |
| 11 | 34 | 11 | 6 | 6 | 0.183 | 3.500 | 1.000 | 0.017 | *Dickinsonia* | *Temnoxa* | Positive | 0.983 |
| 1 | 23 | 5 | 6 | 4 | 0.083 | 1.600 | 0.999 | 0.017 | *Andiva* | *Onega* | Positive | 0.982 |
| 4 | 27 | 4 | 8 | 4 | 0.089 | 1.700 | 1.000 | 0.018 | *Armillifera* | *Parvancorina* | Positive | 0.982 |
| 4 | 35 | 4 | 8 | 4 | 0.089 | 1.700 | 1.000 | 0.018 | *Armillifera* | *Tribrachidium* | Positive | 0.982 |
| 23 | 35 | 6 | 8 | 5 | 0.133 | 2.500 | 0.999 | 0.024 | *Onega* | *Tribrachidium* | Positive | 0.975 |
| 27 | 34 | 8 | 6 | 5 | 0.133 | 2.500 | 0.999 | 0.024 | *Parvancorina* | *Temnoxa* | Positive | 0.975 |
| 34 | 35 | 6 | 8 | 5 | 0.133 | 2.500 | 0.999 | 0.024 | *Temnoxa* | *Tribrachidium* | Positive | 0.975 |
| 5 | 21 | 9 | 4 | 4 | 0.100 | 1.900 | 1.000 | 0.033 | *Beltanelliformis* | *Nemiana* | Positive | 0.967 |
| 2 | 17 | 3 | 7 | 3 | 0.058 | 1.100 | 1.000 | 0.036 | *Anfesta* | *Kimberella* | Positive | 0.964 |
| 3 | 17 | 3 | 7 | 3 | 0.058 | 1.100 | 1.000 | 0.036 | *Archaeaspinus* | *Kimberella* | Positive | 0.964 |
| 13 | 25 | 3 | 7 | 3 | 0.058 | 1.100 | 1.000 | 0.036 | *Grypania* | *Palaeopascichnus* | Positive | 0.964 |
| 17 | 32 | 7 | 3 | 3 | 0.058 | 1.100 | 1.000 | 0.036 | *Kimberella* | *Tamga* | Positive | 0.964 |
| 18 | 25 | 3 | 7 | 3 | 0.058 | 1.100 | 1.000 | 0.036 | *Liulinqitaenia* | *Palaeopascichnus* | Positive | 0.964 |
| 20 | 25 | 3 | 7 | 3 | 0.058 | 1.100 | 1.000 | 0.036 | *Mezenia* | *Palaeopascichnus* | Positive | 0.964 |
| 4 | 10 | 4 | 5 | 3 | 0.055 | 1.100 | 0.999 | 0.037 | *Armillifera* | *Cyanorus* | Positive | 0.961 |
| 11 | 27 | 11 | 8 | 7 | 0.244 | 4.600 | 0.998 | 0.037 | *Dickinsonia* | *Parvancorina* | Positive | 0.961 |
| 1 | 11 | 5 | 11 | 5 | 0.152 | 2.900 | 1.000 | 0.040 | *Andiva* | *Dickinsonia* | Positive | 0.960 |
| 10 | 11 | 5 | 11 | 5 | 0.152 | 2.900 | 1.000 | 0.040 | *Cyanorus* | *Dickinsonia* | Positive | 0.960 |
| 11 | 37 | 11 | 5 | 5 | 0.152 | 2.900 | 1.000 | 0.040 | *Dickinsonia* | *Yorgia* | Positive | 0.960 |
| 17 | 37 | 7 | 5 | 4 | 0.097 | 1.800 | 0.998 | 0.038 | *Kimberella* | *Yorgia* | Positive | 0.960 |
| 23 | 34 | 6 | 6 | 4 | 0.100 | 1.900 | 0.997 | 0.046 | *Onega* | *Temnoxa* | Positive | 0.951 |
| 1 | 5 | 5 | 9 | 0 | 0.125 | 2.400 | 0.022 | 1.000 | *Andiva* | *Beltanelliformis* | Negative | 0.978 |
| 5 | 17 | 9 | 7 | 1 | 0.175 | 3.300 | 0.040 | 0.998 | *Beltanelliformis* | *Kimberella* | Negative | 0.958 |

Table S9: Co-occurrence analysis for the White Sea Russian dataset showing only significant associations.

Sp1_inc is the number of sites which have taxa 1. Obc_cooccur is the observed number of sites with both species. Prob_cooccur is the probability both species occur at a site. Exp_cooccur is the expected number of sites having both taxa. P_Lt probably that the two taxa would co-occur at a frequency less than observed and P_gt is the probability that the two taxa would co-occur at a frequency greater than observed. Difference is the difference between observed and expected probabilities. Where difference > 0.95 the association is considered significant.
