# Supplementary material for: Metacommunity analyses show an increase in ecological specialisation throughout the Ediacaran period
Source: PLoS Biol. 2022 May 17;20(5):e3001289. doi: 10.1371/journal.pbio.3001289 (PMC9113585; doi:10.1371/journal.pbio.3001289)
Supplement: Attachment — Submitted filename: Edenetal2022_response_final.pdf [file pbio.3001289.s019.pdf]

UNIVERSITY OF CAMBRIDGE
Department of Zoology

Dr Emily G. Mitchell
Senior Research Fellow
ek338@cam.ac.uk

4th March, 2022

Dear Dr Roberts,

Thank you very much for the opportunity to revise and resubmit our manuscript, on "*Metacommunity analyses show increase in ecological specialisation throughout the Ediacaran*" (manuscript # PBIOLOGY-D-21-01148R2). We really appreciated the attention to detail, especially and thoroughness of the comments. We have taken on board the suggestions for improvement and clarification. Overall, we believe the manuscript is very much improved and represents a major advance in our understanding of Ediacaran ecology. In addition to the revised manuscript (changes and corrections in colour), we include a point-by-point response to the reviewers' comments.

Yours Sincerely,

Dr Emily Mitchell
on behalf of the authors

Editor:
IMPORTANT: You will see that while the reviewers find the manuscript somewhat improved, they still raise a significant number of concerns that must be addressed. I note a tone of exasperation in their reviews, so please do take this opportunity to make a conscientious effort to thoroughly revise the manuscript and to address these issues; we will only consult the reviewers one more time, and if they still remain dissatisfied then we may decide not to consider the manuscript further.

Our apologises for our oversights. We have addressed all the reviewers' comments as listed below, and have thoroughly revised the manuscript.

The Academic Editor asked me to emphasise the need for sensitivity analysis: "Regarding the need for some type of sensitivity analysis, I fully agree with rev #1. The sensitivity analysis is not complex and certainly will add much more robustness to the analysis and presentation. The problem is that, somehow, the potentially confounding effects of size difference among the assemblages need to be accounted for. Rev #1 gives some cues about how to frame this."

We agree that sensitivity analyses ensure that the results are robust, and so have included new analyses and discussion as follows. Our results show that the differences between the Nama assemblage and the White Sea and Avalon assemblages are the number of non-random co-occurrences and the significance of depth with site score, so these are the two aspects we focused on for our sensitivity analyses. We have also added a paragraph discussing the metacommunity structure of the environmental sub-samples of the Avalon and White Sea assemblages, which have similar sample sizes as Nama, to emphasize what is and isn't a sampling artefact on Lines 423-454.

> *The Nama assemblage has notably less localities (9) than either the Avalon (29) or the White Sea (28), which could suggest that differences in the Nama are merely an artefact of sampling. Therefore, it is important to understand how these sampling differences could affect the EMS analyses and the co-occurrence analyses. We assessed these biases in two ways: 1) by comparing results of environmental subsets of the Avalon and White Sea which are similar in size to the Nama assemblage and 2) by simulating Avalon and White Sea data by sub-sampling the larger datasets to that of Nama – 9 localities then testing for significant non-random co-occurrences and for a correlation between site score and depth.*
>
> *First, in terms of co-occurrence, for the Avalon subsets, the margin slope (23) and outer shelf (6) have 4.5% and 0% significant non-random co-occurrence, and for the White Sea the deep subtidal has 4.1% significant co-occurrences and middle shelf has 7.9%. These values are much smaller than that of the Nama at 16.7%. Furthermore, they show, an increase from the Avalon to the White Sea, thus confirming the overarching pattern of increasing co-occurrences found in the full sets. In terms of metacommunity structure, the Avalon environmental subsets have the same metacommunity strucdfture (nested clumped species loss) as the whole Avalon, with negative turnover and small coherence factors. Similarly, the White Sea environmental subsets both have the same Quasi-Clementsian structure as the whole White Sea assemblage. Thus the changes of metacommunity structure from the Avalon to the White Sea are maintained within the environmental sub-sets with sample sizes similar to those available for Nama.*
>
> *Second, we performed randomized tests for the co-occurrence and depth analyses for the Avalon and White Sea data, subsampling the datasets 1000 times each from 29 (Avalon) and 28 (White Sea) to 9 (Nama). Avalon had significantly less non-random co-occurrences than the While Sea and Nama ($p_{av.vs.ws} = 0.016$; $p_{av.vs.nm} = 0.016$), in contrast to the White Sea, which showed no significant difference in numbers of co-occurrences ($p_{ws.vs.nm} = 0.158$). In order to test for the significance of the depth correlation we performed Spearman's test each of the 1000 sub-sampled data. Only a small number of sub-samples had a significant correlation for depth (24 out 1000 fro Avalon; and 17 out of 1000 for the White Sea). Therefore, we are confident that our results showing an increase in co-occurrence between the Avalon and Nama and an increase in depth structure between the White Sea and Nama are not artefacts of different sampling, but robust signals.*

Reviewer #1:

General

This revision adds a lot of clarity to the initial submission, however I still have a lot of questions that I do not believe are addressed in the current version. There is still some language confusing taxa interactions vs. taxa co-occurrences that need to be addressed,

Our apologies for these mistakes, which we have rectified throughout, as noted below.

and I have also tried to note areas where the text can be improved to clarify the motivation of the contribution. Specifically, I found the section detailing the author's hypothesis testing framework to be inadequate and have tried to offer suggestions on that front. Throughout, and especially in the results, the writing needs work. There are still sentences that aren't following grammatical rules, which I feel - at this level - should have been addressed.

Thank you for these suggestions, which are very much appreciated. We have made the changes as suggested, as detailed below. Our sincere apologies for these mistakes, we have been through the document thoroughly to ensure similar errors do not remain.

I think that the results section could also be better organized to translate the central findings of the paper… perhaps via subheadings.

We agree, and have added subheadings to the results.

Unless I missed it completely, one outstanding question that I have with respect to the analysis as a whole is the effect of community size on the metacommunity analysis. Some of the communities vary a lot in size… Nama is very small; White Sea is very large. Are the analyses sensitive to size? If so, which are, and which are not? It's not really addressed (again, unless I missed it), except to say that some communities were not included because they were *too* small (4)… but what is too small?

Because the difference between the largest (White Sea) and smallest (Nama) is what forms the basis for interpreting whether there was either an extinction or restructuring, understanding what of the results might be influenced by the size difference is *incredibly important* in my view. Incorporating a sensitivity analysis to show that the results are insensitive to community size (hopefully) would be straightforward to do, and I think necessary to disentangle these potential effects. Or perhaps there is a) prior exploration of these effects in previous pubs using this analysis, or b) that White Sea is broken into middle/outer shelves makes the size comparisons more comparable. Yet if the latter is the argument, I would want to understand — looking at Fig 3a — what drives the differences between White Sea (all together) vs. White Sea Middle Shelf, White Sea Outer Shelf, White Sea Deep Subtidal (which look more similar to Nama)… the structure or the size?

Our apologies for not clarifying this point previously. To assess the effect of sampling size on our results we took two approaches. Firstly, by considering how the environmental sub-samples in the Avalon and White Sea (which have similar numbers of localities) differ from the Nama assemblage. The environmental sub-sets largely mirrored the broader assemblage patterns. Secondly, by randomization tests whereby we subsample the Avalon and White Sea data to that of Nama, then use the frequency of how many times the simulations were more than the Nama to indicate significance. For these simulations we addressed the key differences between the White Sea and the Nama, namely the number of significantly non-random co-occurrences and whether the simulated data had a significant correlation with depth. As noted in the manuscript on Lines 423-454, the Avalon had significantly less co-occurrences than the White Sea or Nama. Nama and the White Sea didn't have significantly different co-occurrences. Neither the Avalon and White Sea simulations showed significant correlations with depth:

> *The Nama assemblage has notably less localities (9) than either the Avalon (29) or the White Sea (28), which could suggest that differences in the Nama are merely an artefact of sampling. Therefore, it is important to understand how these sampling differences could affect the EMS analyses and the co-occurrence analyses. We assessed these biases in two ways: 1) by comparing results of environmental subsets of the Avalon and White Sea which are similar in size to the Nama assemblage and 2) by simulating Avalon and White Sea data by sub-sampling the larger datasets to that of Nama – 9 localities then testing for significant non-random co-occurrences and for a correlation between site score and depth.*
>
> *First, in terms of co-occurrence, for the Avalon subsets, the margin slope (23) and outer shelf (6) have 4.5% and 0% significant non-random co-occurrence, and for the White Sea the deep subtidal has 4.1% significant co-occurrences and middle shelf has 7.9%. These values are much smaller than that of the Nama at 16.7%. Furthermore, they show, an increase from the Avalon to the White Sea, thus confirming the overarching pattern of increasing co-occurrences found in the full sets. In terms of metacommunity structure, the Avalon environmental subsets have the same metacommunity strucdfture (nested clumped species loss) as the whole Avalon, with negative turnover and small coherence factors. Similarly, the White Sea environmental subsets both have the same Quasi-Clementsian structure as the whole White Sea assemblage. Thus the changes of metacommunity structure from the Avalon to the White Sea are maintained within the environmental sub-sets with sample sizes similar to those available for Nama.*
>
> *Second, we performed randomized tests for the co-occurrence and depth analyses for the Avalon and White Sea data, subsampling the datasets 1000 times each from 29 (Avalon) and 28 (White Sea) to 9 (Nama). Avalon had significantly less non-random co-occurrences than the While Sea and Nama ($p_{av.vs.ws}$ = 0.016; $p_{av.vs.nm}$ = 0.016), in contrast to the White Sea, which showed no significant difference in numbers of co-occurrences ($p_{ws.vs.nm}$ = 0.158). In order to test for the significance of the depth correlation we performed Spearman's test each of the 1000 sub-sampled data. Only a small number of sub-samples had a significant correlation for depth (24 out 1000 fro Avalon; and 17 out of*

1000 for the White Sea). Therefore, we are confident that our results showing an increase in co-occurrence between the Avalon and Nama and an increase in depth structure between the White Sea and Nama are not artefacts of different sampling, but robust signals.

Specific

L13 - It isn't clear in the abstract that assessing these changes in diversity using an 'Elements of Community Structure' framework is posing an alternative hypothesis to a catastrophic mass extinction

Thank you for pointing out this lack of clarity. We have clarified this point on Lines 13-19:

> *The first animals appear during the late Ediacaran (572 – 541 Ma); an initial diversity increase was followed reduction in diversity, often interpreted as catastrophic mass extinction. We investigate Ediacaran ecosystem structure changes over this time period using the "Elements of Metacommunity Structure" framework to assess whether this diversity reduction in the Nama was likely caused by an external mass extinction, or internal metacommunity restructuring.*

L16 - Again, the data collected cannot say anything about how taxa interact… only how they co-occur

Our apologies, we have corrected this on Line 18: interaction -> *association*.

L79-81 - is there an assumption here related to even dispersal across all localities? If there was uneven dispersal (some localities connected more strongly than others), would this change any of the assumptions in the analyses?

Metacommunity analyses do not assume even dispersal, and there are studies which investigate the effect of differing levels of dispersal on metacommunity structure which show that intermediate levels of dispersal result in the highest levels of community differentiation due to biotic and abiotic effects (Leibold et al. 2004). In cases where dispersal is limited, this is associated with nested metacommunities i.e. negative turnover (Henio et al. 2014). In our analyses, the only assemblage that exhibited negative turnover was the Avalon assemblage, so the most likely to be subject to dispersal limitation. These Avalonian localities are the most geographically close, occurring within the same basin (Boddy et al. 2021). As such, they are relatively highly connected compared to the White Sea or Nama assemblages, which are biogeographically more scattered, and so a dispersal limitation is unlikely to be the driving force behind these Avalonian results.

This point has been added on Lines 81-84

EMS does not assume even dispersal across all sites, with intermediate levels of disturbance associated with the highest levels of filtering of community by biotic and abiotic factors (29), and dispersal limitation associated with negative Turnover (33).

L84 - it should be mentioned here that these metrics are hierarchical rather than independent

Agreed, and added in on Line 91-93

Three metacommunity metrics are calculated to determine the structure: Coherence, Turnover, and Boundary Clumping (29–31), which are hierarchical rather than independent of each other.

L86 - From Figure 1, I only count 9… I get that the others are the Quasi/Mixed structures, but it is hard to glean from the figure. More generally I don't really understand the quasi structures…

Our apologies for the confusion about the number of different possible structures – there is some variation in the literature to whether checkerboard, random and quasi structures are included. There are two non-positive-coherence options, checkerboard and random (2), then 6 positive coherence structures and then 6 quasi structures, total 14. We have ensured that we consistently refer to this number throughout on Lines 94, 191, 287 and 559. On the suggestion of Reviewer 2 we have added their Table (Table S1) explaining the structures to the supplementary to further clarify this point.

Figure 1 - I find the orientation hard to follow… aside from the (-)(+)(0) layout (I'll let that one go), on the left negative boundary clumping is on top and positive boundary clumping is below; on the right, positive boundary clumping is on top, and negative boundary clumping is below. For the reader to keep all of these attributes in their heads, these stylistic choices can make the paper much harder to follow. There is also an errant 'minus sign' just below 'TURNOVER'… not sure if it's supposed to be there or not?

Our apologies for this confusion. We have reformatted this figure, to show the positive, negative or not significant values in red, blue and grey. We have changed the structure to be consistent with both positive and negative turnover.

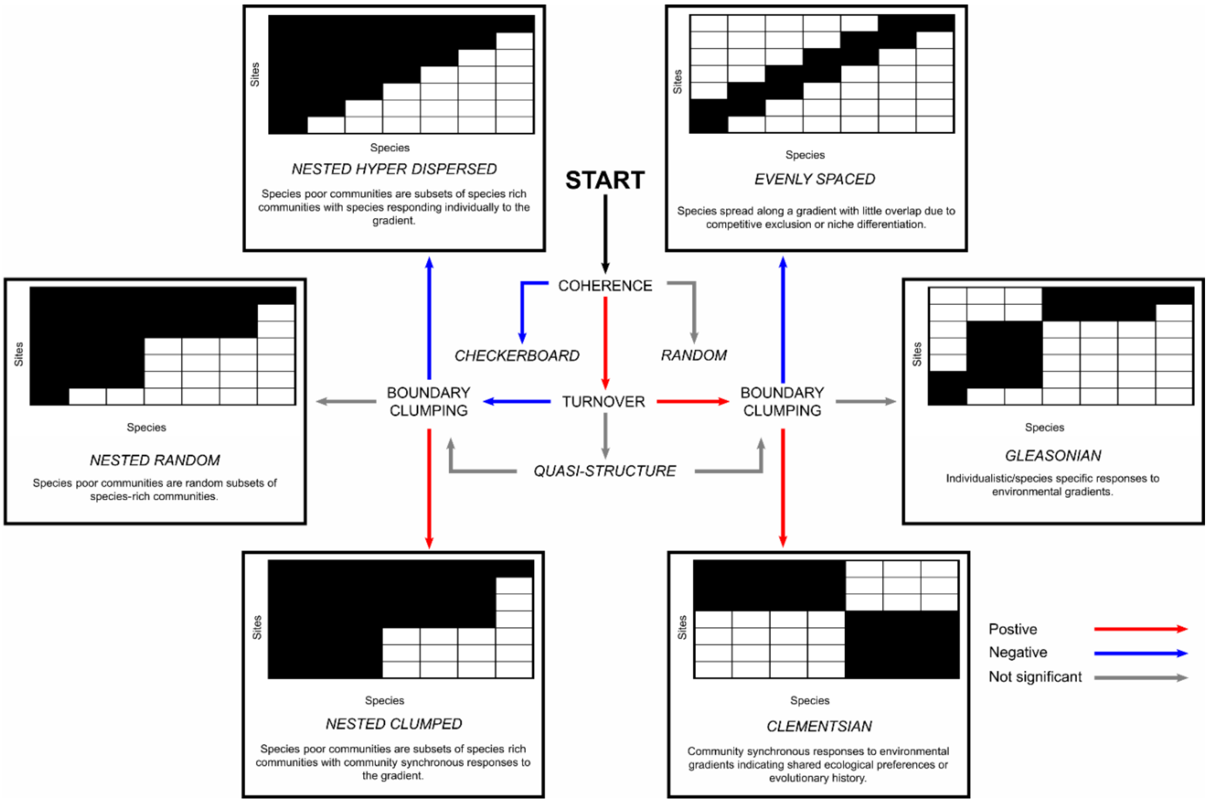

Intro - this is a nice layout - I like how the first aim is very community structure oriented, the second aim is community v environment, and the third aim is species pairwise correlations… it might be worthwhile to emphasize this tiered approach to the analysis, as I see it as a core strength of this contribution (just a suggestion).

Thank you for your kind comments.

L149 - a strange sentence: "because co-occurence does not necessarily correspond to interaction, here we interpret pair-wise interaction…" do you mean pair-wise correlations?

Our apologies, we have corrected this point on Line 163:

…here we interpret pair-wise interactions correlations (or associations) to…

L153-159 - This section is strangely worded… it is stated that 3 hypotheses will be tested, but proposing a hypothesis test is different than guessing what outcome will occur from an analysis. For example, the current statement goes like this: you 1) hypothesize that increased taxonomic diversity is reflected in terms of increased taxa co-occurrences, 2) hypothesize that Ediacaran data exhibits strong metacommunity structure, and 3) will test whether there is evidence of an extinction… But in reality, you are attempting to *build support* for an alternative hypothesis for extinction… To be more rigorous about this, it seems to me that you are testing between 3 hypotheses:

1) Null: nothing happened (or the signal can't be distinguished from random)
2) Alt Hypothesis 1) an externally-caused mass extinction happened

3) Alt Hypothesis 2) an internally-caused restructuring happened.

You are proposing that a) increased ecological complexity via co-occurrences and b) strong metacommunity structure will lend support to Alt Hypothesis 3, and against 1 and 2 (I presume the Null has already been refuted in prior work showing the diversity drop, etc). Alternatively, if your metacommunity structure analysis supports negative Turnover, that would be support for Alt Hypothesis 2. If I am accurately describing the intent of this paper, it is not very well captured by the organization in Lines 153-160, so I would suggest being a bit more clear about what alternative hypotheses you are testing against, rather than stating guesses at what the results will be.

Thank you for these suggestions. We have incorporated the previous predictions and the hypotheses as follows on, Lines 167-179.

> *Based on the literature, we can make predictions about how we may expect metacommunity structure to change throughout these Ediacaran assemblages. We predict that the increase in taxonomic and morphological diversity between the Avalon and White Sea assemblages (5) is reflected in more ecological complexity in terms of increased taxa co-occurrences. We predict that the total set of Ediacaran data exhibits strong metacommunity structure which reflects the previously recovered assemblages (7–9) and that the influence of environmental gradients increases between the Avalon and White Sea assemblages (18). Finally, we will use these analyses to test between three hypotheses relating to the underlying causes behind the White Sea – Nama drop in taxonomic diversity: 1) Null hypothesis: Changes in taxonomic diversity are not present, or are not detectable, 2) External mass-extinction: test whether there is evidence of a catastrophic extinction event between the White Sea and Nama (9,22,46). Such an event would lead to negative turnover, so a nested metacommunity structure (40), or 3) Internal-restructuring: increased ecological complexity via co-occurrences and strong metacommunity leading to stronger niche partitioning.*

L177 - I would explicitly state what you are pooling… here it is presence/absence data of organisms as a function of locality.

Thank you for the suggestion, which we have implemented on Lines 198-199:

> *First, we analysed all the presence/absence data of organisms as a function of sites irrespective of their assemblage, in order to test whether the assemblage definitions represented distinct communities.*

L220 - I don't think the z-score is a measure of statistical significance… it is standardized deviance away from the mean… so it is telling us how similar or different each community is from the average across communities in units of standard deviation (i.e. 1 standard deviation away from the mean, etc), if I am understanding it correctly. Echoing my comment in the first draft, this needs to be clearly explained.

We have expanded on our explanation of the z-score on Lines 96-101:

> *To determine whether an observed metric score differs significantly from random, we computed the z-score, which measures its distance from the mean of the randomisations (simulation mean) as the number of standard deviations (thus making it comparable across metrics with difference units). If the z-score is negative, the observed value is smaller than the simulated mean, if it is positive, then it is greater than the simulated mean; $z \geq |3|$ indicates a significant deviation.*

L228 - should this be referring to Fig 3? I don't get any insight to Coherence/Boundary Clumping from looking at Fig 2

Our apologies for this typo, which is now corrected Fig. 2 -> Fig. 3.

Generally - what is the effect of size differences between communities? The Nama community is very small and the White Sea community is very big. Are measures of Metacommunity Structure sensitive to size heterogeneity? If so, how could this skew your perspective of these systems? One way to examine this… if you took random subsamples of taxa from the White Sea, where the number of subsamples is equal to that of the Nama community, measured metacommunity structure, and repeated this many many times, do you get measurement distributions with a mean similar to that using the full dataset?

The impact of sample size on our results has been expanded on in two ways: by considering the environmental subsamples as well as randomization analyses of the effect on sample size on our key results. These discussions show the relative robustness of sample sizes for our results, as detailed on Lines 423-454:

> *The Nama assemblage has notably less localities (9) than either the Avalon (29) or the White Sea (28), which could suggest that differences in the Nama are merely an artefact of sampling. Therefore, it is important to understand how these sampling differences could affect the EMS analyses and the co-occurrence analyses. We assessed these biases in two ways: 1) by comparing results of environmental subsets of the Avalon and White Sea which are similar in size to the Nama assemblage and 2) by simulating Avalon and White Sea data by sub-sampling the larger datasets to that of Nama – 9 localities then testing for significant non-random co-occurrences and for a correlation between site score and depth.*
>
> *First, in terms of co-occurrence, for the Avalon subsets, the margin slope (23) and outer shelf (6) have 4.5% and 0% significant non-random co-occurrence, and for the White Sea the deep subtidal has 4.1% significant co-occurrences and middle shelf has 7.9%. These values are much smaller than that of the Nama at 16.7%. Furthermore, they show, an increase from the Avalon to the White Sea, thus confirming the overarching pattern of increasing co-occurrences found in the full sets. In terms of metacommunity structure, the*

Avalon environmental subsets have the same metacommunity strucdfture (nested clumped species loss) as the whole Avalon, with negative turnover and small coherence factors. Similarly, the White Sea environmental subsets both have the same Quasi-Clementsian structure as the whole White Sea assemblage. Thus the changes of metacommunity structure from the Avalon to the White Sea are maintained within the environmental sub-sets with sample sizes similar to those available for Nama.

Second, we performed randomized tests for the co-occurrence and depth analyses for the Avalon and White Sea data, subsampling the datasets 1000 times each from 29 (Avalon) and 28 (White Sea) to 9 (Nama). Avalon had significantly less non-random co-occurrences than the While Sea and Nama ($p_{av.vs.ws} = 0.016$; $p_{av.vs.nm} = 0.016$), in contrast to the White Sea, which showed no significant difference in numbers of co-occurrences ($p_{ws.vs.nm} = 0.158$). In order to test for the significance of the depth correlation we performed Spearman's test each of the 1000 sub-sampled data. Only a small number of sub-samples had a significant correlation for depth (24 out 1000 fro Avalon; and 17 out of 1000 for the White Sea). Therefore, we are confident that our results showing an increase in co-occurrence between the Avalon and Nama and an increase in depth structure between the White Sea and Nama are not artefacts of different sampling, but robust signals.

L306 - strangely worded… it begins as a question but without a '?' ends as a statement.

Our apologies, we have re-worded the sentence to clarify our point, Lines 341-346:

The 11/32 positive associations that differed between the middle shelf and deep subtidal environments show that the community associations are not consistent between the subsets and the assemblage as a whole. The underlying processes that contribute to these differences in both inter-taxa interactions and environmental factors could be due to organism behavioural plasticity, leading to different behaviours in different environments.

The Results section could really use some editing… there are still quite a few sentences that are poorly constructed, and it overall feels meandering. I had a hard time understanding what the important results were relative to the less-important findings. Easy to get lost in this section. Perhaps some reorganization would help with subheadings? There was also a lot of discussion material in the results section, which I don't personally mind, though it made a discussion section feel superfluous.

Thank you for the suggestion of subheadings, which we have added Line 196: *Total Ediacaran taxaset*; Line 255 *Avalon metacommunity analysis*; Line 323; *White Sea metacommunity analyses* Line 370*: Nama metacommunity analyses* and Line 423: *Effect of sampling biases.*

We included some more discursive elements in our results section to help understand the processes behind each individual assemblage, so that the discussion could focus on the overall patterns across the assemblages, rather than the individual metacommunities.

L397 - Again (and again), these results cannot say anything about 'interactions between taxa'… only associations

We apologise for the times throughout the manuscript when we made this mistake, namely conflating significant co-occurrences with interactions. We have corrected it throughout (Lines 18, 162, 297, 299, 301, 303, 304, 344, 474, 493. However, we feel this a one of the few places where it is appropriate, since we are saying that significant co-occurrences can be the result of species interactions or habitat associations. As such, while the other instances are of course changed, we feel this one should remain.

L423-424 - This is a big leap. While there is definitely work showing that generalists may have a selective advantage in the face of large extinction events, it is quite a thing to say that the presence of generalists would imply a catastrophic extinction, or that their absence is incongruent with an extinction!

We agree that it is a big leap, but in our case it is just one of several lines of evidence. We have clarified why we think our results are inconsistent with a mass extinction on Lines 497-519, namely the presence of generalists, positive (not negative) turnover, and an increase in niche specialization.

A White Sea – Nama catastrophic environmental extinction is in consistent with our results for three reasons. Firstly, a catastrophic mass extinction implies that surviving taxa within the Nama assemblage are more likely to be generalists (8,9,65), contrary to our results. We have shown an increased influence of paleoenvironment and niche specialisation with the Nama metacommunity showing significant correlation between community composition and depth, in contrast to the White Sea and Avalon metacommunities. Second, the Nama metacommunity exhibits a non-significant but positive turnover, indicating more turnover along the gradient (more niche differentiation). If the Nama assemblage metacommunity structure was due to underlying extinction/colonisation dynamics, we expect to see an increase in nestedness (40), as indicated by negative turnover, contrary to our positive Nama turnover (Table 1). Thirdly, this increase in turnover suggests not only higher ecosystem complexity but also increased taxa specialisation and narrower niche breadths is coupled with an increase in within-community structuring between the White Sea and Nama assemblages, with an slight increase in non-random taxa associations (16.1% to 16.7%). A decrease in taxonomic and morphological diversity in the Nama (23) may reflect that, within this assemblage, Ediacaran organisms show significant palaeoenvironmental preferences, and thus reduced environmental tolerances, resulting in multiple different types of mutually exclusive communities, each of which exhibits a simple structure within its narrow niche (23). An increase in within-community structure in the form of ecosystem engineering (66) and reef complexity (20) provides supporting evidence that despite a decrease in taxonomic diversity, the Nama assemblage represents an ecological development from the White Sea assemblage, not a recovery from a catastrophic extinction event. Our results are further supported by birth-death models of stem and crown group diversification, which predict Ediacaran-like diversification patterns for bilaterians and produce patterns which can be easily mistaken for mass extinctions (67).

Reviewer2

As a reviewer of the original submission, I am glad to have had the opportunity to provide a review of the revised manuscript of this paper. I thought the original paper had promise, but with numerous structural issues, which the other reviewers also recognized. I was pleased to see that they have attempted to address all the issues raised by the reviewers. The current manuscript is a marked improvement as a result.

That said, the current version still has numerous issues that will require at least one more round of revisions – some of these should have been caught by a careful re-reading prior to resubmission- so that is disappointing. I will try to enumerate these by Line. I will also make a suggestion that I hope will improve the clarity of the paper.

Our apologies for our oversights. Thank you for your suggestions and detailed comments, which we have addressed below.

1. Line 44. Is "the reduction in taxonomic diversity" different from "diversity drop"? Not clear!

Our apologies for the lack of clarity, which we have rectified Lines 46-47:

> *This reduction in taxonomic diversity, sometimes referred to as the "diversity drop",*

2. Lines 73, 89. Both start with "First." Reading further, the one on Line 89 should be deleted.

Thank you for spotting this mistake, which we have now rectified.

3. Lines 73-87. Although there is evidence for larval transport and low provinciality, that does not indicate to me that these can all be treated as a metacommunity in the sense that ecologists would use it. Instead, acknowledge this but point out that for the purpose of this paper we can treat them as metacommunities.

We agree this is important, and so acknowledged this point, and also expanded on how dispersal limitation impacts the EMS analyses on Lines 77-84

> *Most fossil metacommunities do not fulfil the requirements of random sampling that would be needed to analyse them with such an ecological framework. However due to their exceptional preservation, the Ediacaran metacommunities are an exceptional census of the benthic assemblages present at the time, making them amenable to be analysed within the EMS framework. We note that EMS does not assume even dispersal across all sites, with intermediate levels of disturbance associated with the highest levels of filtering of community by biotic and abiotic factors (29), and dispersal limitation associated with negative Turnover (33).*

4. Line 86-127.

a. I went back to the original paper by Presley et al. (ref. 30) and found that there were actually 14 metacommunity types, if one includes Checkerboard and Random. Each of the six categories with positive coherence and significant positive or negative turnover has a non-significant, quasi- equivalent.

Our apologies to the confusing surrounded the number of metrics, which comes from some variation in the literature about whether to include quasi structures and/or checkerboard and random coherence. We have ensured that we consistently refer to 14 structures number throughout on Lines 94, 191, 287 and 559.

b. Even with the revised figure caption and text, I was still confused about the relationships among the metrics and the community types. I was especially confused by what was meant by a "quasi-" structure and what it implied about the metacommunity. Given that many of the analyses were consistent with a "Clementsian quasi-structure," this needed further discussion.

We have expanded on what quasi-structures are on Lines 120-123:

Quasi-structures have the same fundamental characteristics as the idealized structures, but because range turnover is not significantly different from random, it is likely that the underlying structuring mechanisms are weaker than those for which turnover is significant.

c. I have made a table (see below) because it clarified for me the properties of the various metacommunity types. I suggest using something like this in the next revision.

Thank you for providing this table, which we have added to the supplementary information to aid understanding of the EMS, and references on Lines 193 and 325. We feel between the updated figure and your table, the structure should be understandable to the reader.

d. I share with one of the other reviewers the issue of capitalization of the metrics. I advise not capitalizing them after they are introduced, and leave the capitalization to the metacommunity types, which are really what the paper should be about. As a comparison, "mean" and "standard deviation" are not capitalized.

We have de-capitalized the metrics throughout, for example for Turnover-> turnover on Lines 115, 118, 120, 122, 127, 133, 135, 141, 177, 190, 191, 206. 237, 259, 326, 373, 501, 204, 505, 555, 563.

e. Lines 115-124 do not belong here! They are a fragment from the results (Lines 304..) (a careful readover should have caught this – do not make this the reviewers job!)

Our apologies for not catching this, we agree we should have caught it previously, and it is now removed.

5. Lines 139-142 and Figure 2. Not an accurate description of reciprocal averaging (correspondence analysis). The method ordinates the samples (sites) by their species composition (variable); it also ordinates the species by which site they are in. Variable ordination scores are averages of the case ordination scores and case ordination scores are averages of the variable ordination scores, "thus "reciprocal averaging." Both plot on the same axes. Depth is an independent variable, which can then be plotted against the RA axes to help with the interpretation. So, are the axes of Figure 2 are probably the scores for the sites and species on the first RA axis; the left-hand plot of depths is independent data, not used to do the ordination.

Thank you for your help with these descriptions. We have attempted to rewrite this paragraph order to ensure the descriptions are correct on Lines 146-156:

Secondly, we used Spearman rank correlations to test whether within-assemblage community composition is correlated with depth. The ordering of the sites was given by the ordination output from the EMS analyses (Fig. 2), which is produced by reciprocal averaging, a type of correspondence analysis which ordinates the sites (y-axis of Fig. 2) based on their species composition (x-axis of Fig. 2) (31). This ordering groups the sites together with similar community composition, and we can see from Figure 2 that the assemblages (indicated by different colours) are grouped together, and that the depths (shown alongside the y-axis) show a correspondence with these assemblages, with the Avalon sites deeper, then increased shallowing up the y-axis and ordination with the Nama sites being the most shallow. This first-axis ranking of the sites was then used to test whether there was a significant association with depth.

Also: make clear in caption to Figure 2 that this a reciprocal averaging ordination.

We have added this point on Line 227, and think that the above paragraph will help to further emphasize this point.

Sites are ranked based on reciprocal averaging ordination.

6. Line 172. Again, there are 14, not 12 models, all six illustrated models have quasi-equivalents

Our apologies for this mistake which has now been rectified 12->14.

7. Lines 177-186. This paragraph badly needs rewriting.

a. On Line 178, you point to Figure 2, but then say nothing at all about it and then jump right away into Figure 3 and then back to Table 1. There needs to be a detailed explanations of both Figure 2 and Table 1, including what is meant by site scores.

We have rewritten the paragraph below, expanding on what is happening in Figure 2 and Table 1.

First, we analysed all the presence/absence data of organisms as a function of sites irrespective of their assemblage, in order to test whether the assemblage definitions represented distinct communities. The sites were ranked using reciprocal averaging ordination (y-axis of Fig. 2), which provided an ranking which was consistent with previous work that grouped communities into the Avalon, White Sea and Nama assemblages (Waggnor 2003; Boag et a. 2016). The coherence, turnover and boundary clumping values were calculated, and the simulated mean was used to determine if that score was significantly different (Table 1). Table 1 gives the score, simulated mean and significant level for each of the total set, Avalon, White Sea and Nama assemblages, and environmental subsets of the assemblages. When analysing the total dataset, we found positive coherence, turnover and boundary clumping, characteristic of a Clementsian structured metacommunity (Fig. 3a, Table 1). Site ordination scores were significantly associated with assemblage (H=57.7, df=2, p<0.001, SI Table 4), indicating strong compositional difference among the assemblages. Site scores were also significantly correlated with depth (ρ = -0.427, p<0.001, Table 2), suggesting that the structuring in the dataset may be due to the differences in depth between sites. However, since depth also

significantly varies with assemblage (H=54.0, df=2, p<0.001, Table 2), it is not possible to tell whether the structuring is due to depth or another factor that varies with assemblage.

b. Looking at the results, it is clear that the relationship between depth and scores only holds true for the Nama, so that it will strongly influence the pooled results.

We would disagree for two reasons. Firstly, the strong relationship between the depth and assemblage has been established by earlier work such as Waggnore 2003; Boag et al. 2016). Because the different assemblages are largely from different depths, we would expect there to be a relationship between assemblage (which is strongly correlated with the order ranking) and depth in our results too. Secondly, the Nama assemblage is only 9 out of a total of 127 sites, so that even if the depth-score relationship was strong, it would be unlikely to be strong enough to have a significant effect across the whole dataset, since it comprises of only 7.1% of the total number of sites.

8. Lines 188-190. How does the low level of non-random associations compare to what is observed in modern communities or other paleontological examples? Is this unusually low or high? See: LYONS, S. K et al.. 2016. Holocene shifts in the assembly of plant and animal communities implicate human impacts. Nature, 529, 80-83.

Thank you for drawing our attention to this interesting paper. We have added how it compares to our results on Lines 459 – 461, where we point out that while our Ediacaran results are lower than most extant examples, there are examples with similar numbers. Because most of the examples used are not marine, we feel that detailed comparisons are limited use, so we think the below provides the right balance between context and comparison.

The total set of Ediacaran data exhibits strong metacommunity structure, consistent with previous analyses that resolve multiple assemblages (7–9). Both the total dataset and the individual assemblages have relatively low numbers of non-random co-occurrences (9.8% - 16.7%) compared to many extant analyses (e.g. 35-63% (61,62)), as well as terrestrial fossil communities (such as averaging 64% aggregated pairs from the Carboniferous to the Holocene, and 37% from the Holocene to the present (63), although percentages of non-random co-occurrences are similar to at least some extant benthic communities (16.3%) (63).

9. Line 211-212. What is the simulated mean value of the metric? Not discussed in the text.

We have added an explanation of the simulated mean on Lines 96-100:

To determine whether an observed metric score differs significantly from random, we computed the z-score, which measures its distance from the mean of the randomisations as the number of standard deviations (thus making it comparable across metrics with difference units). If the z-score is negative, the observed value is smaller than the simulated mean, if it is positive, then it is greater than the simulated mean; $z \geq |3|$ *indicates a significant deviation.*

10. Lines 256-259. How are the succession stages determined? Needs more detail.

The succession stages were determined by largely qualitative interpretations of ordination plots and community compositions by Clapham et al. 2003. We have explained this on Lines 276-278.

Differences in Avalonian community composition have been suggested to represent different stages of community succession, based on community parameters, cluster analyses and MDS ordination (13)

11. Lines 290, 334 – this is why there needs to be a more detailed explanation of quas-structures!

We have added in a description of quasi-structures on Lines 120-123, alongside the new Table S1 which explains these in more detail:

Quasi-structures have the same fundamental characteristics as the idealized structures, but because range turnover is not significantly different from random, it is likely that the underlying structuring mechanisms are weaker than those for which turnover is significant.

12. Line 334 – so this is the same structure as the White Sea?

Yes, and to emphasize this point we have added Lines 373-374:

…. the same metacommunity structure as the White Sea assemblage

13. Line 395-396. Is this a significant increase, given the large number of random associations? Again, we need context in terms of other communities.

Our randomization analyses comparing the White Sea to the Avalon find a significant difference between the number of co-occurences, so we have added this *p*-value on Lines 470,

….results in an increase of non-random taxa associations from the Avalonian biota (9.8%) to the White Sea biota (16 .1% , p_{avalon} = 0.016).

Lines 447-448:

Avalon had significantly less non-random co-occurrences than the While Sea and Nama (both p_{avalon} = 0.016),

14. Line 423-426. I am confused here. Based on table 1, the White Sea, except for the outer shelf, and the Nama have the same structure. The change is from the Avalon.

Yes, the largest change occurs between the Avalon and White Sea, and the White Sea and Nama have the same metacommunity structure. We have expanded out this paragraph to make it clearer why our results are not consistent with a mass extinction on Lines 497-519:

A White Sea – Nama catastrophic environmental extinction is contrary to our results for three reasons. Firstly, a catastrophic mass extinction implies that surviving taxa within the Nama assemblage are more likely to be generalists (8,9,65), contrary to our results. We have shown an increased influence of paleoenvironment and niche specialisation

with the Nama metacommunity showing significant correlation between community composition and depth, in contrast to the White Sea and Avalon metacommunities. Second, the Nama metacommunity exhibits a non-significant but positive turnover, indicating more turnover along the gradient (more niche differentiation). If the Nama assemblage metacommunity structure was due to underlying extinction/colonisation dynamics, we would expected to see an increase of nestedness (40), as indicated by negative turnover, contrary to our positive Nama turnover (Table 1). Thirdly, this increase in turnover suggests not only higher ecosystem complexity but also increased taxa specialisation and narrower niche breadths coupled with an increase in within-community structuring between the White Sea and Nama assemblages, with an slight increase in non-random taxa associations (16.1% to 16.7%). A decrease in taxonomic and morphological diversity in the Nama (23) may reflect that, within this assemblage, Ediacaran organisms show significant palaeoenvironmental preferences, and thus reduced environmental tolerances, resulting in multiple different types of mutually exclusive communities, each of which exhibits a simple structure within its narrow niche (23). An increase in within-community structure in the form of ecosystem engineering (66) and reef complexity (20) provides supporting evidence that despite a decrease in taxonomic diversity, the Nama assemblage represents an ecological development from the White Sea assemblage, not a recovery from a catastrophic extinction event. Our results are further supported by birth-death models of stem and crown group diversification, which predict Ediacaran-like diversification patterns for bilaterians and produce patterns which can be easily mistaken for mass extinctions (67).

15. Line 468… this is Methods and should be so indicated.

We have split the Materials and Methods under two subheadings, Materials on Line 528 and Methods on Line 553.

16. Lines 469-487. This is confusing; both the RA and metacommunity analyses are being discussed in the same paragraph. Break these apart and make sure the RA is carefully described.

We have expanded on our explanation of RA in the main text, Lines 546-551:

Secondly, we used Spearman rank correlations to test whether within-assemblage community composition is correlated with depth. The ordering of the sites was given by the ordination output from the EMS analyses (Fig. 2), which is produced by reciprocal averaging, a type of correspondence analysis which ordinates the sites (y-axis of Fig. 2) based on their species composition (x-axis of Fig. 2) (31). This ordering groups the sites together with similar community composition, and we can see from Figure 2 that the assemblages (indicated by different colours) are grouped together, and that the depths (shown alongside the y-axis) show a correspondence with these assemblages, with the Avalon sites deeper, then increased shallowing up the y-axis and ordination with the Nama

sites being the most shallow. This first-axis ranking of the sites which were then used to test whether there was a significant association with depth.

Then we clarified these analyses on Lines 554-558:

The R package Metacom was used for the EMS analyses (37). The first step of the EMS analyses was to use reciprocal averaging to ordinate the sites based on their species composition (31, Fig. 2). Metacommunity structure is then quantified based on this ordering, via the calculation of the three metrics related to metacommunity structure: coherence, turnover and boundary clumping (37).
