# Supplementary material for: Metacommunity analyses show an increase in ecological specialisation throughout the Ediacaran period
Source: PLoS Biol. 2022 May 17;20(5):e3001289. doi: 10.1371/journal.pbio.3001289 (PMC9113585; doi:10.1371/journal.pbio.3001289)
Supplement: Attachment — Submitted filename: Edenetal2021_response_final.docx [file pbio.3001289.s017.docx]

**Dr Emily G. Mitchell**

Senior Research Fellow

ek338@cam.ac.uk

14^th^ October, 2021

Dear Dr Roberts,

Thank you very much for the opportunity to revise and resubmit our manuscript, on "*Metacommunity analyses show increase in ecological specialisation throughout the Ediacaran*" (manuscript # PBIOLOGY-D-21-01148R1). All of the reviews were very useful, and we really appreciated the attention to detail, especially and thoroughness of the comments. We have taken on board the suggestions for improvement and clarification. Overall, we believe the manuscript is very much improved and represents a major advance in our understanding of Ediacaran ecology. In addition to the revised manuscript (changes and corrections in colour), we include a point-by-point response to the reviewers’ comments.

Yours Sincerely,

Dr Emily Mitchell

on behalf of the authors

*****************************************************
REVIEWERS' COMMENTS:


Reviewer #1:

The authors present here a series of analyses of presence/absence data for 124 taxa across 86 Ediacaran localities to examine whether a large drop in diversity in the late Ediacaran was due to an external disturbance (mass extinction) or the results of eco/evolutionary forces such as niche specialization and competition restructuring the communities. The authors claim that their analyses point to the latter, and while I don't have strong reason to doubt this claim, the conclusions that they draw from their analyses are very hard to follow. In large part this is due to the fact that they a) don't adequately describe the methods they use, and b) don't clearly point the reader to how changes in one metric or another leads to different interpretations and why this should be the case. In part this can be helped by porting some of the basic explanations of what is being done and why to the fore of the manuscript, with details in the methods of course, but even the methods are
lacking in terms of specifics. While I understand that the analyses were created and reported elsewhere, I think it is important for the basics to be reported here - especially for the 3 metrics used to parse potential community organizations that they are examining. Apart from the minimal methods, I found the objectives of the paper to be hard to follow. Really what the manuscript needs most is motivation. Why is it important to examine potential changes in community structure, and why are the methods employed the appropriate ones to use in this case? For example - the authors state on L58 "we will investigate the structuring mechanisms within these assemblages". While structure is clearly assessed, it is much less clear how the authors extract *mechanism* from differences in measured structural attributes. While the authors may be quite justified in their interpretation of mechanism, it needs to be clearly stated, which it currently is not. As it stands there are so many
unclear aspects of the manuscript, I find it impossible to judge in terms of its technical merits.

We apologise of the lack of clarity within the manuscript, and have expanded the methodology and added in further explanation of the analyses we performed and provided more ecological background for the different EMS metrics. These changes are listed below in response to direct points.

We agree with reviewer one that we need to take more care regarding mechanisms. We have made the following changes to the manuscript:

Line 65: *mechanisms -> structural attributes*

Line 408: *mechanisms -> taxa associations.*

My interpretation then is that this paper has a lot of potential - it is an interesting look at a very old community (well, communities). The conclusions that the authors describe are also very interesting as they suggest that a previously interpreted mass extinction may in fact be due to expected changes in the eco/evolutionary trajectories of these communities without such a dramatic externality such as a mass extinction to bring about community change. The pieces are all in place, but the authors need to spend a little more effort to connect them, in my humble opinion. I'd be excited to review an revision that incorporates some of these - mainly textual rather than technical - changes. I was also a bit surprised at the number of grammatical errors and incomplete sentences throughout the manuscript, as if things were quickly shuffled around prior to submission. I'd advise the authors to make make sure that all of their sentences follow grammatical rules during revision and
that sentence structure is complete. On a more minor note, ensure that American/British spellings are corrected - both 'specialisation' and 'specialization' are used. Not sure which Plos prefers.

Our apologies for these mistakes and errors which we have now rectified throughout, using British spellings as per other published PLoS papers.

L19 - The authors might consider making it more clear that this is a reinterpretation of the 'diversity drop' described in the first sentence of the abstract, rather than an independent (and not necessarily mutually exclusive) finding.

Agreed and changed Line 13:

*drop, interpreted -> drop, often interpreted*

L21 - Moreover, I think that it is more correct to say that this 'diversity drop' can be explained by an alternative hypothesis (structuring and resulting competitive exclusion, etc)... it is not certain it is one or the other (mass extinction), correct?

Agreed, we have expanded on this Lines 47-53:

*“This diversity drop has been suggested to be caused by either an environmental driven catastrophic environmental extinction or biotic replacement driven extinction (9,23,24,21). Recent work has shown that a biotic replacement driven extinction, whereby mobile metazoans out-competed soft-bodied Ediacaran organisms through bioturbation and ecosystem engineering, is unlikely due in part to prolonged co-occurrence of trace fossils with soft-bodied biota (1,25). Other currently unknown and/or unpreserved intrinsic causes behind a biotic replacement model cannot not be excluded at the moment.”*

L51-53 - I'm not sure what 'using modularity to compare' means... modularity is a network property, but it may be more correct to say that they found differences in the compartmentalization of XXX in the networks rather than to say they use the metric to compare (as it's not a robust statistic for directly comparing networks on its own). Also it is a bit jargony the way it is used here, so some clarification would help. The term 'cluster' used on L53 is also not clear in the way it is used, as it is stated it is a prior analysis, but does this mean it is the same analysis as the one that employs modularity? Overall, the description of the prior work - both the means and the primary conclusions - need to be clarified. It is also important to specify what network is being used. I assume co-occurrence?

Our apologies for the lack of clarity, which we have corrected on Line 58:

*The network structures of Ediacaran… -> The network structures of the co-occurrence of Ediacaran…*

Line 60:

*these prior cluster and network analyses-> However, the prior cluster analyses of Refs (7,8,10) and network analyses of Ref 9 have….*

L59 - A detail, but 'using a matrix' is less clear than the fact that the authors compiled presence/absence for 86 localities, 124 taxa (wow!)... Whether or not you put this information into a matrix format is less material... If this matrix format is important for the EMS framework, I would suppose it's organization is also important, so should be described a bit more clearly (there are many ways this information can be put into a matrix).

Agreed, and corrected on Line 67:

“presence-absence data encompassing 86 Ediacaran”

L67-68 - these metrics need to be defined. They can mean different things in different contexts, and it should be assumed that the reader is not familiar with the EMS framework.

L69 - what does 'fits within the EMS framework' mean? Is there a statistical measure whereby these metrics tell us whether the inputted species across localities are functioning as a spatial meta community? It would be more helpful - rather than to refer to the 'EMS framework' - to tell the reader what the methods employed are telling us ecologically. In other words, I care less about the framework than I care about what the framework tells us regarding the ecology, so the ecology should be put center stage, with the framework  being a means to an end.

We agree with both these points and so have moved text from the methods and SI as well as expanding the background on these metrics and their ecological understanding Lines 89-138:

*First, Coherence is a measure of the extent to which all the taxa respond to the same environmental gradient, where this gradient may result from the interplay of several biotic and abiotic factors that differ between sites (33). Coherence is positive when the taxa in the site-by-taxa matrix all respond to the same environmental gradient. Most extant well-sampled metacommunities display significant positive Coherence due to similarities in evolutionary history, ecological preferences or life-history trade-offs within communities (33). A significant negative Coherent site-by-taxa matrix reflects a high number of mutually exclusive taxa pairs creating checkerboard patterns	(7,9,10). These checkerboard patterns do not have further underlying structure (in contrast to positive coherence patterns, Fig. 1), as there is no* *discernible gradient to which all the taxa respond. Negative co-occurrences and significant segregation/checkerboard patterns can be formed from strong competition, grazing/herbivory or strongly non-overlapping niches, all of which form similar metacommunity patterns due the presence of mutually exclusive pairs of taxa (29,31,33). A non-significant Coherence reflects no significant metacommunity structuring (Fig. 1). For metacommunities which have positive Coherence, the Turnover	metric tests the amount of taxa replacement between sites (34). If taxa ranges are nested within each other, there is less Turnover than expected by chance along the gradient (significant negative). If there are more differences in site taxa composition along the gradient than expected by chance, Turnover is significantly positive and the structure is non-nested. Non-significant Turnover indicates a weaker structuring mechanism, termed a quasi-structure (Fig. 1) (30). The final metric, Boundary Clumping measures the extent to which taxa range limits cluster at the same sites across the environmental gradient (33). The range limits can be clumped (significant positive), hyperdispersed (significant	negative) or random (non-significant).*

*Positive Coherence and negative Turnover result in nested metacommunities with taxa-poor sites being predictable sub-sets of taxa-rich sites, implying that species are dispersal limited (35). Nested metacommunities have been shown to be associated with a low degree of spatial connectivity and environmental variation (35) and have been shown to govern post-extinction dynamics (36). Depending on Boundary Clumping, taxa loss can be clumped (leading to a Nested Clumped community), hyper-dispersed (Nested Hyper-dispersed community), or random (Nested Random Community, Fig. 1) (13). Clumped species boundaries tend to be associated with the transitions between different biomes, where two biological communities mix, in contrast to hyper-dispersed species loss where species loss is evenly distributed across the range (30). Positive Coherence and Turnover with hyper-dispersed (negative) Boundary Clumping describes an Evenly Spaced metacommunity (Fig. 1). Where Coherence, Turnover and Boundary Clumping are all positive, the metacommunity is classed as Clementsian (Fig. 1), where groups of taxa with similar range boundaries co-occur and respond in a similar way to environment gradients (33,37). Taxa within Clementsian metacommunities respond synchronously to environmental gradients, suggesting physiological or evolutionary trade-offs associated with environmental thresholds (38), tend to result from high levels of environmental variability and spatial connectivity (35), and are found to be the most common (e.g. (39)). When Coherence and Turnover are positive but there is no significant Boundary Clumping, the metacommunity is described as Gleasonian (Fig. 1) where each taxon reacts individualistically to environmental gradients (30).*

Figure 1 - Cool figure, and I think I get the descriptions of the outcomes, but the mechanisms by which these outcomes supposedly derive are completely mysterious. Also - I can't seem to find what NS means… Non-structured? Another very odd thing about this figure is that - for example - coherence is split from left to right as (-) (+) (0)… the other metrics are presented in various order from left to right. Because these metrics are continuous variables, this swapping around in various ways is confusing. If there is a way to order the resulting community structures in a way that makes sense with respect to the continuous nature of the variables?

The figure is adapted from Presley et al. 2010, so we have kept the order to ensure consistency. To include clarify in the figure we have expanded the caption Lines 163-175:

*Figure 1: Idealised metacommunity structures adapted from Ref (30).*

*These graphs show taxa abundance patterns in idealised metacommunities of several taxa (represented by different colors) which respond to a latent environmental gradient (they exhibit significant positive Coherence). The first step of the analyses (START) is to determine whether the metacommunity exhibits positive, negative or random Coherence. Random Coherence corresponds to no significant (NS) metacommunity structure, negative Coherence is a checkerboard pattern (46), so significant mutual exclusivity between species and sites. Positive Coherence indicates mutual co-occurring taxa associations and there are several different possible models. For positive Coherence, Turnover and Boundary Clumping are calculated to determine the type of metacommunity structure. Non-significant Turnover corresponds to quasi-structures. These EMF analyses enable the structure of metacommunities to be grouped into one of 12 models: 1) Random, 2) Checkerboard, 3-5) Nested clumped, random and hyperdispersed, 6-8) Clementsian, Gleasonian and evenly spaced, 9-10) Quasi nester clumped and hyper-dispersed and 11-12) Quasi Clementsian and evenly spaced.*

L70-72 - Can you describe what you mean by random or non-random? There are many ways by which species could be randomly distributed across sites. In the case of this test, what does random mean. More importantly, what does random and non-random imply eco/evolutionarily. The reader needs to be pointed to what these different conditions mean in terms of our interpretation of the community.

We have expanded our explanation on Lines 166-172:

*The first step of the analyses (START) is to determine whether the metacommunity exhibits positive, negative or random Coherence. Random Coherence corresponds to no significant (NS) metacommunity structure; negative Coherence is a checkerboard pattern (43), so significant mutual exclusivity between species and sites. Positive Coherence indicates mutual co-occurring taxa associations and there are several different possible models. For positive Coherence, Turnover and Boundary Clumping are calculated to determine the type of metacommunity structure. Non-significant Turnover corresponds to quasi-structures.*

L72 - what is reciprocal averaging? Averaging of what?

We have explained this on Lines 140-143:

*Secondly, to test whether within-assemblage community composition is correlated with depth, we use reciprocal averaging, a type of correspondence analysis which ordinates the sites based on their community composition (31), and then correlates the first-axis ranking (i.e. the order of sites based on community composition) with depth.*

L67-74 - Both in the abstract and later in the paper, a central point appears to be posing eco/evo restructuring of the meta community as an alternative hypothesis for late Ediacaran diversity declines. If that is a central aim, I would think it would be mentioned here? Overall I think the aims need to be motivated by how they will be filling in knowledge gaps… i.e. you are listing what you intend to do, but not 'why'.

We have expanded the introduction to give more context for our work on lines 38-42:

*The White Sea assemblage (558-550 Ma) shows a large increase in morphological diversity, including putative bilaterians (5), in tandem with a greater ecological diversity which includes the appearance of grazing, herbivory and wide-spread motility (15,16). These innovations are coupled to the development of dense communities with high community heterogeneity between environments (16,17) and increased taxa sensitivity to fine-scale environment (12,18).*

Lines 47-53:

*This diversity drop has been suggested to be caused by either an environmental driven catastrophic environmental extinction or biotic replacement driven extinction (9,23,24,21). Recent work has shown that a biotic replacement driven extinction, whereby mobile metazoans out-competed soft-bodied Ediacaran organisms through bioturbation and ecosystem engineering, is unlikely due in part to prolonged co-occurrence of trace fossils with soft-bodied biota (1,25). Other currently unknown and/or unpreserved intrinsic causes behind a biotic replacement model cannot not be excluded at the moment.*

And on what we are testing Lines 154-161:

*Taken together these analyses enable us to test three hypotheses: We hypothesise that the increase in taxonomic and morphological diversity between the Avalon and White Sea assemblages is reflected in more ecological complexity in terms of increased taxa co-occurrences. We hypothesise that the total set of Ediacaran data exhibits strong metacommunity structure which reflects the previously recovered assemblages (7–9) and that the influence of environmental gradients increases between the Avalon and White Sea assemblages(18). Finally, we will use these analyses to test whether there is evidence of a catastrophic extinction event between the White Sea and Nama (9,22,42). Such an event would lead to negative Turnover, so a nested metacommunity structure (36).*

Regarding this and prior comments on clarity, I understand that the details are in the Materials and Methods, but the Intro/Results/Discussion shouldn't be totally mysterious and jargony. The material needs to be introduced and described in enough detail for the reader to understand why things are being done and what this will mean, even if they will later need to dig into the methods to get the details. So far, I am helplessly adrift as I read through the intro, and I don't know why things are being done and for what reason. Apart from an illusion in the abstract, I'm not even clear that you are testing an alternative hypothesis for the decline in late Ediacaran diversity. The Intro really needs to be re-envisioned to emphasize the aims of the paper, and the motivation for applying the analyses that are described... and what this can perspective can shed light on. [Circling back after reading the materials and methods, they give me a sense of what the measures are
measuring, but I don't have a sense of the details. Things like z-scores, which are reported, are not mentioned].

We have expanded the methods and materials as detailed above (and addressed through comments below) so believe we have now provided sufficient context for our work.

L93-96 - This is a cool finding!

Thank you.

Table 1 - It would be very helpful to orient the readers - in a very basic way - how changes in the three metrics are used to identify the different listed interpretations... i.e. High coherence and low turnover and clumping lead to XXX communities... Low coherence and high turnover lead to YYY communities, etc. Also need to define in Layman's terms what coherence/turnover/clumping means in this context. Not dissimilar from what is written on L155-157, but as a roadmap for the different potential end-conditions in Figure 1. [Circling back, this is done a bit in materials and methods - some of this needs to be placed in the fore, so that the reader can follow what changes in these metrics represent in terms of community structure]

We believe that with our newly expanded background and methodology this context is now provided.

Figure 3 - I can find the (general) definition for boundary clumping (though I think all metrics: coherence, turnover, boundary clumping should be quantitatively defined here - otherwise I have no way of understanding how or why different sites have the values they do), but I have no idea what a 'clump' is, as 1, 3, 6 clumps are reported in Figure 3. Even more problematically, nowhere in the manuscript can I find reference to the z-value displayed in the figure. Because only the raw values of coherence, turnover, and boundary clumping are discussed… with important meanings for (+,-,0), it would be helpful to report why it is preferable to look at the z-values (presumably z-scores?). For the z-scores, what is serving as the mean and sd used to compute values for individual sites? In other words, is the Avalon z score: (Avalon x - Avalon mean) / Avalon sd? Or is it (Avalon x - Total mean)/(Total sd)?

We have modified the figure to remove the confusing “clumps” and so that the value of Boundary clumping is now provided by the radius of the circles.

We have clarified this in the caption Lines 223-224.

*The size of the circle represents the value of the Boundary Clumping score.*

The Z-value is the Z-score, which we have rectified in the figure and explained on Lines 221-222.

*The z-score indicates the statistical significance.*

We have clarified this in the methods and materials section on Line 455:

*calculated using a z-test (i.e. calculating the z-score) of the observed*

We have clarified how the z-score is calculated on Lines 476-477:

*For each sub-section of data (e.g. each assemblage), the z-score is calculated relative to that sub-set only, rather than the total set of data*.

L142 - Wait... what are the 12 models? Either I missed it or it is not mentioned prior to this reference, which is confusing and a bit disconcerting.

There are 12 possible models, which are detailed in figure 1, and expanded on in the caption Lines 172-175:

*These EMF analyses enable the structure of metacommunities to be grouped into one of 12 models: 1) Random, 2) Checkerboard, 3-5) Nested clumped, random and hyperdispersed, 6-8) Clementsian, Gleasonian and evenly spaced, 9-10) Quasi nester clumped and hyper-dispersed and 11-12) Quasi Clementsian and evenly spaced.*

L168-171 - Not to be a stats snob, but is this significantly different in a statistical sense? Not everything needs a p-value, but you might consider a different adjective if you use significantly different in a statistical sense elsewhere.

Agreed, and we have changed significant to “notably” where statistical tests have not been used on Line 278 and to “notable” on Line 303.

L171 - I think this is a stretch... you don't have interaction information, just co-occurrence information which may or may not (often not) correlate with interactions. I think what is needed here is an argument that the occurrence in different communities implies a change in interactions between species. I would think you could only truly support this if species A is in a completely unique environment where there are no other co-occurring species between the communities being compared. Then species A must be interacting with completely different species in each site. If there are even a small number of co-occurring species in 2 communities with species A, species A might not be changing its behavior - it may just have stronger interactions with the subset of species that co-occur in both sites. So some consideration of the co-occurrence vs. interaction concepts is important here.

We have modified the paragraph to more accurately represent our results Lines 306-317:

*Several taxa seemed to have unique taxa associations in each subdivision despite very similar community composition (Fig. S4. Tables S6-7), which naively we would expect to lead to the same taxa associations.* *So, what could underpin the 11/32 positive associations that differ between the middle shelf and deep subtidal environments? Variation in both inter-taxa interactions and environmental factors can cause organisms to exhibit behavioural plasticity, acting differently in different environments. Alternatively, differences in taxa associations for a given taxon may reflect the inclusion of several taxa with different environmental preferences and behaviors within one taxonomic group (e.g. Dickinsonia). However, as most Ediacaran taxonomic groupings are monotypic, the differences in taxa associations in different environments are more likely due to plastic responses to, for example, variation in resource limitation or the presence of different competitors or ecosystem engineers.*

L197 - very interesting result!

Figure 4 - this is such a cool figure

Thank you for your appreciative comments.

L222 - A period missing between 'depth' and 'Claudina'?

Corrected Line 369.

L240 - Seems like not a full sentence - something appears to be missing as the next sentence discusses a 'final radiation' but no context wrt earlier radiations.

Our apologies for these mistakes. We have deleted the half sentence so it is no longer at the start of the discussion. We have clarified the radiations on line 325: ‘second-wave radiation’,

*“where the first-wave radiation was the “Avalon Explosion”* *(5,45,52).”*

L247 - Is complexity here being used as a synonym for high community heterogeneity between environments?

We have clarified what we mean by complexity: Lines 399-402.

*Further evidence for this increased ecological complexity (i.e. greater interactions and associations between taxa) is provided by the fact that the Avalonian assemblage had minimal environmental structuring, whilst we detected both Gleasonian and quasi-Clementsian metacommunity structure depending on the palaeoenvironments for the White Sea assemblage.*

L256 - Because this is all inference, I would recommend connecting this inferred mechanism to the pattern that your data bear out...

We have clarified that we are discussing taxa associations, not mechanisms Lines 408-409:

*“With organisms exhibiting different taxa associations in different environments across a wide range of depths.”*

L269 - the use of 'autecological' and 'synecological' strategies/interactions is used only once. I would suggest using less jargony terms or at least define them. Seems unnecessary given the terms don't appear anywhere else.

Agreed, and corrected Lines 421-423:

*Thus, we have shown that the increase in complexity of the taxon-specific ecological strategies utilised throughout the Ediacaran is mirrored in the complexity of the community interactions.*

L282 - is Turnover capitalized for a reason?

It is capitalised to ensure the reader is clear we are referring to the metric, rather than more general ecological turnover. If it is more confusing than helpful, we are happy to change this.

L336 - would checkerboard patterns be random if there is mutual exclusivity? Seems not possible. I would expect more 'randomness' when coherence = 0? I.e. no significant structuring, which implies non-structuring, which I imagine here as somewhat random?

We have clarified checkerboard patterns in Lines 95-100:

*These checkerboard patterns do not have further underlying structure (in contrast to positive coherence patterns, Fig. 1), as there is no discernible gradient to which all the taxa respond. Negative co-occurrences and significant segregation/checkerboard patterns can be formed from strong competition, grazing/herbivory or strongly non-overlapping niches, all of which form similar metacommunity patterns due the presence of mutually exclusive pairs of taxa (29,31,33).*

L342 - This implies that Turnover is only defined for coherence > 0? Is that right?

Correct. Our expanded explanation of the metrics now makes this clear on Lines 102-105:

*“the Turnover metric tests the amount of taxa replacement between sites (37). If taxa ranges are nested within each other, there is less Turnover than expected by chance along the gradient (significant negative). If there are more differences in site taxa composition along the gradient than expected by chance, Turnover is significantly positive and the structure is non-nested. Non-significant Turnover indicates a weaker structuring mechanism, termed a quasi-structure (Fig. 1) (30).”*

L349 - I think how 'significance' is calculated in each of these contexts needs to be described here.

Significance is defined using z-scores as described on Lines 475-477:

*“statistical significance is calculated using a z-test (i.e. calculating the z-score) of the observed absences to embedded absences in randomized null matrices (31). For each sub-section of data (e.g. each assemblage), the z-score is calculated relative to that sub-set only, rather than the total set of data.”*

L351-361 - some of these definitions and verbiage would be much more helpful in the fore-part of the manuscript.

L363 - This is really a personal decision, but because you present 3 primary metrics that form the heart of your manuscript, I would at least consider reporting how they are calculated in the materials and methods unless it is unduly complicated. This would help 'self-contain' the manuscript a bit more and make everything much easier to understand without having to bounce to other publications to gain a deeper understanding of what is going on.

We agree and have moved sections to the main section of the manuscript (noted in green) and as detailed above.

References - A lot of output that is not in journal format - need curated.

Our apologies, these have been rectified.

Reviewer #2:

Metapopulation and metacommunity concepts have long been recognized by paleontologists   (see, for example, papers in M. McKinney and J. Drake (eds.), 1998. Biodiversity Dynamics). In many ways they are an excellent model for paleontological "communities," which generally can be both spatially and temporally averaged and thus probably represent samples from a regional species pool, rather than a snapshot of a single living community. In this paper, the authors apply the metacommunity concept to Ediacaran assemblages across a wide spatial, temporal, and geographic scale. In particular, they apply the "Elements of Metacommunity Structure" framework, using an R package to analyze the data. There results suggest that the various Ediacaran assemblage show a range of metacommunity structures and are suggestive of an increase in niche specialization over time.

Overall I enjoyed reading this; I have not read about metacommunities in some time and was very pleased to be reintroduced to the concept and to see that it being actively used within paleontology. As far as I know, the use of the Elements of Metacommunity Structure and the mathematical applications of it are novel in paleontology; certainly to the study of the enigmatic Ediacaran assemblages. The figures are very nicely done. There are, however, some questions and suggestions I have before I could recommend publication:

1.      Leibold et al. 2004 define "a metacommunity as a set of local communities that are linked by dispersal of multiple potentially interacting species." Given that that the Ediacaran sites within each defined assemblage ("community') are widely separated in space and time, the authors need to discuss how the metacommunity concept applies in this context. Why is it valid to treat these as metacommunities, since there is no evidence they are linked by dispersal?

The connectivity of Ediacaran communities is well established in the literature. To emphasize this important point we have explained this on lines 74 – 81:

*“Ediacaran communities vary in how much they are separated in time and space, from ecological to geological time-scales (8,9,13), and their organisms have been shown to have large dispersal ranges based on reproductive mode (28,32,33) and species occurrence over large space and time scales (34). Because the connectivity of these Ediacaran communities via dispersal has been established, here we define metacommunities as sets of fossil localities (communities) which are connected by the dispersal of many species (29).*”

2.      Figure 1, and much of the results section, make no sense unless one first jumps to the Materials and Methods.
Although I understand that the journal requires that this section come at the end, that is not necessary for the discussion of the theoretical framework. The material on lines 328-361 needs to be merged with the  Introduction section. The description of the data and of the R package can be kept in Materials and Methods.

Thank you for the suggestion. We have substantially increased the method explanations in the main text, as per above and Lines 73-161.

*“First, we will use the “Elements of Metacommunity Structure” (EMS) framework to investigate emergent properties of groups of connected communities that may arise from taxa interactions, dispersal, environmental filtering and the interaction of these factors (29–31) (Fig. 1). Ediacaran communities vary in how much they are separated in time and space, from ecological to geological time-scales (8,9,13), and their organisms have been shown to have large dispersal ranges based on reproductive mode (28,32,33) and species occurrence over large space and time scales (34). Because the connectivity of these Ediacaran communities via dispersal has been established, here we define metacommunities as sets of fossil localities (communities) which are connected by the dispersal of many species (29). The EMS framework is a hierarchical analysis which identifies properties in site-by-taxa presence/absence matrices which are related to the underlying processes shaping taxa distributions (31), but to date has limited application to the fossil record (35). Three metacommunity metrics are calculated to determine the structure: Coherence, Turnover, and Boundary Clumping (29–31). The values and statistical significance of these metrics determine where the metacommunity fits within the 12 different metacommunity types within the EMS framework (Fig. 1), with different metric combinations indicating different underlying processes behind the metacommunity structure.*

*First, Coherence is a measure of the extent to which all the taxa respond to the same environmental gradient, where this gradient may result from the interplay of several biotic and abiotic factors that differ between sites (36). Coherence is positive when the taxa in the site-by-taxa matrix all respond to the same environmental gradient. Most extant well-sampled metacommunities display significant positive Coherence due to similarities in evolutionary history, ecological preferences or life-history trade-offs within communities (36). A significantly negatively Coherent site-by-taxa matrix reflects a high number of mutually exclusive taxa pairs creating checkerboard patterns (7,9,10). These checkerboard patterns do not have further underlying structure (in contrast to positive coherence patterns, Fig. 1), as there is no discernible gradient to which all the taxa respond. Negative co-occurrences and significant segregation/checkerboard patterns can be formed from strong competition, grazing/herbivory or strongly non-overlapping niches, all of which form similar metacommunity patterns due the presence of mutually exclusive pairs of taxa (29,31,36). A non-significant Coherence reflects no significant metacommunity structuring (Fig. 1). For metacommunities which have positive Coherence, the Turnover metric tests the amount of taxa replacement between sites (37). If taxa ranges are nested within each other, there is less Turnover than expected by chance along the gradient (significant negative). If there are more differences in site taxa composition along the gradient than expected by chance, Turnover is significantly positive and the structure is non-nested. Non-significant Turnover indicates a weaker structuring mechanism, termed a quasi-structure (Fig. 1) (30). The final metric, Boundary Clumping measures the extent to which taxa range limits cluster at the same sites across the environmental gradient (36). The range limits can be clumped (significant positive), hyperdispersed (significant negative) or random (non-significant).*

*Positive Coherence and negative Turnover result in nested metacommunities with taxa-poor sites being predictable sub-sets of taxa-rich sites, implying that species are dispersal limited (38). Nested metacommunities have been shown to be associated with a low degree of spatial connectivity and environmental variation (38) and have been shown to govern post-extinction dynamics (39). Several taxa seemed to have unique taxa associations in each subdivision despite very similar community composition. (Fig. S4. Tables S6-7), which naively we would expect to lead to the same taxa associations. So, what could underpin the 11/32 positive associations that differ between the middle shelf and deep subtidal two environments? Variation in both inter-taxa interactions and environmental factors can cause organisms to exhibit behavioural plasticity, acting differently in different environments. Alternatively, differences in taxa associations for a given taxon may reflect the inclusion of several taxa with different environmental preferences and behaviors within one taxonomic group (e.g. Dickinsonia). However, as most Ediacaran taxonomic groupings are monotypic the differences in the behavior of the taxa associations in different environments are more likely due to plastic responses to, for example, variations in resource limitation or the presence of different competitors or ecosystem engineers.*

*Clumped species boundaries tend to be associated with the transitions between different biomes, where two biological communities mix, in contrast to hyper-dispersed species loss where species loss is evenly distributed across the range (30). Positive Coherence and Turnover with hyper-dispersed (negative) Boundary Clumping describes an Evenly Spaced metacommunity (Fig. 1). Where Coherence, Turnover and Boundary Clumping are all positive, the metacommunity is classed as Clementsian (Fig. 1), where groups of taxa with similar range boundaries co-occur and respond in a similar way to environment gradients (36,40). Taxa within Clementsian metacommunities respond synchronously to environmental gradients, suggesting physiological or evolutionary trade-offs associated with environmental thresholds (41), tend to result from high levels of environmental variability and spatial connectivity (38), and are found to be the most common (e.g. (42) ). When Coherence and Turnover are positive but there is no significant Boundary Clumping, the metacommunity is described as Gleasonian (Fig. 1) where each taxon reacts individualistically to environmental gradients (30).*

*Secondly, to test whether within-assemblage community composition is correlated with depth, we use reciprocal averaging, a type of correspondence analysis which ordinates the sites based on their community composition (31), and then correlates the first-axis ranking (i.e. the order of sites based on community composition) with depth.*

*Thirdly, we will test to determine which pair-wise taxa co-occurrences are significantly non-random, and whether any non-random co-occurrences are positive or negative. We use a combinatorics approach to test whether species pairs are randomly distributed among sites (43). If co-occurrences are significantly non-random, this suggests a shared underlying ecological or evolutionary process. While the interpretation of co-occurrence data is complicated because co-occurrence does not necessarily correspond to interaction (44), here we interpret pair-wise interpretations to within the wider EMS framework where co-occurrences are not taken necessarily as direct taxa interactions, but could also indicate taxa environmental associations and/or disassociations.*

*Taken together these analyses enable us to test three hypothesises: We hypothesise that the increase in taxonomic and morphological diversity between the Avalon and White Sea assemblages is reflected in more ecological complexity in terms of increased taxa co-occurrences. We hypothesise that the total set of Ediacaran data exhibits strong metacommunity structure which reflects the previously recovered assemblages (7–9) and that the influence of environmental gradients increases between the Avalon and White Sea assemblages(18). Finally, we will use these analyses to test whether there is evidence of a catastrophic extinction event between the White Sea and Nama (9,22,45). Such an event would lead to negative Turnover, so a nested metacommunity structure (39).”*

While this is being done, a clearer explanation of the concept of "clumped species loss" is needed; I read line 135-137 several times and I was still left confused (needed to return to the original cited papers). Is that different from "nested species loss" (caption to figure 3)?

We apologise for this confusion, and have changed “clumped species loss” to “nested clumped species loss” on lines 230 and 245. Nested species loss refers to all negative Turnover models (i.e. random, clumped or hyper-dispersed).

3.      In Figure 1, the central region with "start" needs to be explained in the caption. This also seems to be redrawn from the Presley et al. (2010) paper, which needs to be cited here,

Our apologies for missing this Presley et al reference, which we have now added. We have also clarified the caption on lines 163-175.

*“Figure 1: Idealised metacommunity structures adapted from Ref (30).*

*These graphs show taxa abundance patterns in idealised metacommunities of several taxa (represented by different colors) which respond to a latent environmental gradient (they exhibit significant positive Coherence). The first step of the analyses (START) is to determine whether the metacommunity exhibits positive, negative or random Coherence. Random Coherence corresponds to no significant (NS) metacommunity structure, negative Coherence is a checkerboard pattern (46), so significant mutual exclusivity between species and sites. Positive Coherence indicates mutual co-occurring taxa associations and there are several different possible models. For positive Coherence, Turnover and Boundary Clumping are calculated to determine the type of metacommunity structure. Non-significant Turnover corresponds to quasi-structures. These EMF analyses enable the structure of metacommunities to be grouped into one of 12 models: 1) Random, 2) Checkerboard, 3-5) Nested clumped, random and hyperdispersed, 6-8) Clementsian, Gleasonian and evenly spaced, 9-10) Quasi nester clumped and hyper-dispersed and 11-12) Quasi Clementsian and evenly spaced.”*

4.      A small point: lines 110-111, 115 "significant p-values highlighted in bold." Significant at what level? To be frank I think simply giving the p-value is sufficient; there are a great many publications that suggest we move away from the concept of "significant."

We agree, and have removed this comment and un-bolded the p-values.

5.      Lines  129-130: although there are more positive than negative associations, they are both far exceeded by the number of non-associations. This needs to be discussed.

Agreed, that’s a very good point, and we have expanded on it on Lines 387-396:

*The total set of Ediacaran data exhibits strong metacommunity structure, consistent with previous analyses that resolve multiple assemblages (7–9). Both the Total dataset and the individual assemblages have relatively low numbers of non-random co-occurrences (9.8% - 16.7%) compared to many extant analyses (e.g. 35-63% (57,58)), although percentages of non-random co-occurrences are similar to at least some extant benthic communities (16.3%) (59). Previous spatial analyses of Avalonian communities have revealed limited inter-specific interactions (11,14) and limited environmental associations between taxa within communities (12), so the large number of non-significant correlations within the Avalon assemblage are consistent with previous work. Given that the oldest assemblage is dominated by non-co-occurrences, it follows that the subsequent development of Ediacaran metacommunities would have to build from this point and so are not immediately comparable to extant communities with longer evolutionary histories.*

6.      Line 173-174. There is no discussion of taphonomic differences among the Ediacaran sites.  This is a critical issue that needs to be considered.

We agree that it is important to point out that the results are unlikely to be due to taphonomic differences. Ediacaran communities are highly unusual in the fossil record because they consist of almost entirely soft-bodied organisms preserved in-situ. Taphonomic differences between sites have been discussed and analysed in detail in e.g. Boag et al. 2016, Muscente et al. 2019 (referenced on line 34), who found no significant taphonomic influence. We have emphasised this key point by adding the line in the introduction Lines 68-70:

*Ediacaran fossils are commonly found preserved in-situ, so their bedding-planes (that is the rock surfaces that preserve the fossils) preserve near-compete censuses of the communities (15,27,27).*

7.      Line 240. An incomplete sentence.

Our apologies, we have re-worked the section and removed the incomplete sentence.

Reviewer #3:

I apologize for my delay in reviewing the manuscript due to personal issues. I read with interest the manuscript, which explores a key problem in the interface of macroevolution and ecology. Below, I list my comments on points that I think the authors need to address to improve their manuscript.

My comments:

1.      Although the introduction is well written I missed a clear statement of the hypothesis and predictions the authors are testing in this manuscript. For example, Figure 1 describes what I think are hypotheses about metacommunity organization. Having said that, these hypotheses are not proper described in the introduction - the authors only describe what they want to measure but not how these measurements will be used to test predictions of distinct hypothesis.

We have added in a paragraph clearly stating our hypotheses on Lines 154-161:

*Taken together these analyses enable us to test three hypothesises: We hypothesise that the increase in taxonomic and morphological diversity between the Avalon and White Sea assemblages is reflected in more ecological complexity in terms of increased taxa co-occurrences. We hypothesise that the total set of Ediacaran data exhibits strong metacommunity structure which reflects the previously recovered assemblages (7–9) and that the influence of environmental gradients increases between the Avalon and White Sea assemblages(18). Finally, we will use these analyses to test whether there is evidence of a catastrophic extinction event between the White Sea and Nama (9,22,42). Such an event would lead to negative Turnover, so a nested metacommunity structure (36).*

2.      On Figure 1: The laconic legend to figure makes very hard to follow it. This figure could be the heart of the manuscript but its complexity, associated with the minimalist legend imperil its usefulness for the current draft.

We have expanded on Figure 1 caption Lines 163-175.

*“Figure 1: Idealised metacommunity structures adapted from Ref (30).*

*These graphs show taxa abundance patterns in idealised metacommunities of several taxa (represented by different colours) which respond to a latent environmental gradient (they exhibit significant positive Coherence). The first step of the analyses (START) is to determine whether the metacommunity exhibits positive, negative or random Coherence. Random Coherence corresponds to no significant (NS) metacommunity structure, negative Coherence is a checkerboard pattern (46), so significant mutual exclusivity between species and sites. Positive Coherence indicates mutual co-occurring taxa associations and there are several different possible models. For positive Coherence, Turnover and Boundary Clumping are calculated to determine the type of metacommunity structure. Non-significant Turnover corresponds to quasi-structures. These EMF analyses enable the structure of metacommunities to be grouped into one of 12 models: 1) Random, 2) Checkerboard, 3-5) Nested clumped, random and hyperdispersed, 6-8) Clementsian, Gleasonian and evenly spaced, 9-10) Quasi nester clumped and hyper-dispersed and 11-12) Quasi Clementsian and evenly spaced.”*

3.      The estimating of interactions by co-occurrences is a critical assumption of the manuscript. Recently, the work by Dominique Gravel and others criticized the use of co-occurrences to estimate patterns of interaction. Although I understand the limitations of estimating patterns of interaction in fossil record, I think the authors need to at least discuss these limitations in their manuscript.

We agree, and so are careful throughout the manuscript to not infer interaction from association, and have expanded on this issue on Lines 147-152:

*“If co-occurrences are significantly non-random, this suggests a shared underlying ecological or evolutionary process. While the interpretation of co-occurrence data is complicated because co-occurrence does not necessarily correspond to interaction (41), here we interpret pair-wise interactions to within the wider EMS framework where co-occurrences are not taken necessarily as direct taxa interactions, but could also indicate taxa environmental associations and/or disassociations.”*

4.      The use of concepts. There are a few places in the manuscript that the use of the language could be more precise and accurate.  For example, in the abstract middle metacommunity may create confusion between middle referring to space or time. Accordingly, in figure 1, stable communities to the description of gleasonian metacommunities may imply these communities are less stable, what is not necessarily true. These communities are just the outcome of species-specific responses to environmental gradients. Please consider to double check the use of these terms in the manuscript.

We agree, and have checked the use of early, middle, late as well as stable throughout the manuscript and made the following changes:

Line 16:

*“Structuring increased in the second oldest metacommunity,….”*

We have corrected the comment on Gleasonian metacommunities within Figure 1 to:

*Gleasonian: Individualistic/species specific responses to environmental gradients.*

*We have clarified that we are talking about early, middle and late successional stages on Lines 257 – 260*

*“This model is further supported by the co-occurrence analyses which found a negative association between late-succession and early-stage taxa (Charnia and Pectinifrons), and positive associations between late-stage (Primocandelabrum and Charnia), middle-stage (Bradgatia and Charniodiscus) and early-stage (Beothukis and Fractofusus) stage taxa (Fig. S2. Table S3) (13).”*

*Line 274*

*“characteristic of late and middle stage succession..”*

*Line 279*

*“middle and late stage succession sites in..”*

5.      Almost all positive pairwise associations are within assemblages and all negative pairwise associations are between assemblages. Assemblages, in turn, are associated with environmental factors. Thus, I would conclude that there is no need to invoke ecological interactions in shaping patterns of co-occurrence. Or, alternatively, that it is impossible to detangle the effects of environmental gradient from the effects of competition limiting occurrence of species to competitive refugia - since it is of course impossible to perform manipulative experiments. So, I think I am missing something when authors mention the role of competition structuring Ediacarian metacommunities

The points in the manuscript where we refer to interspecific competition we are referring to previous work (Mitchell and Kenchington 2018, Mitchell and Butterfield 2018 and Mitchell et al. 2019) which assessed competition vs environment using spatial point process analyses. In this study, we interpreted the metacommunity results in light of this previous work, rather than using these analyses to do so. We have clarified this point on lines 239-241:

*“The predominance of positive over negative taxa co-occurrences is consistent with previous studies of detailed within-community spatial analyses of focal taxa which found little evidence for lateral resource competition between Avalonian taxa (11,18).”*

And Lines 253-254

*“However, the lack of inter-specific competition found in previous studies using spatial analyses within communities of early-stage communities (11,12,14) suggests that..”*

6.      Please consider defining assemblage, community, and metacommunity in our manuscript to avoid confusion.

We have defined these Lines 80-81:

*here we define metacommunities as sets of fossil localities (communities) which are connected by the dispersal of many species (29).*

7.      There are some descriptions of tables and figures (e.g., lines 108-111) that should be moved to legend of the table/figure.

We have expanded on Figure 1 legend to include the definitions of the different metrics Lines 163-175

*“Figure 1: Idealised metacommunity structures adapted from Ref (30).*

*These graphs show taxa abundance patterns in idealised metacommunities of several taxa (represented by different colors) which respond to a latent environmental gradient (they exhibit significant positive Coherence). The first step of the analyses (START) is to determine whether the metacommunity exhibits positive, negative or random Coherence. Random Coherence corresponds to no significant (NS) metacommunity structure, negative Coherence is a checkerboard pattern (46), so significant mutual exclusivity between species and sites. Positive Coherence indicates mutual co-occurring taxa associations and there are several different possible models. For positive Coherence, Turnover and Boundary Clumping are calculated to determine the type of metacommunity structure. Non-significant Turnover corresponds to quasi-structures. These EMF analyses enable the structure of metacommunities to be grouped into one of 12 models: 1) Random, 2) Checkerboard, 3-5) Nested clumped, random and hyperdispersed, 6-8) Clementsian, Gleasonian and evenly spaced, 9-10) Quasi nester clumped and hyper-dispersed and 11-12) Quasi Clementsian and evenly spaced.”*

In Figure 3 we have expanded the legend to include what the z-score is and what the Circles indicate in terms of Boundary clumping, Lines 220-226:

*“Figure 3. Metacommunity analyses and co-occurrence matrices for each assemblage. a, Metacommunity plot shows a summary of the metacommunity analyses. The z-score indicates the statistical significance. Nested species loss is shown by an open circle; Clementsian by a closed circle; Gleasonian by a black ringed circle. The size of the circle represents the value of the Boundary Clumping score. The Avalonian outer shelf had a random structure and so is not shown. Co-occurrence matrices for b, the Avalon metacommunity, c, the White Sea metacommunity and d, the Nama metacommunities. Positive associations are blue, negative associations are yellow.”*

We have also expanded on the meaning behind the metrics in the main text on Lines 89-138 to help clarify them.

*“First, Coherence is a measure of the extent to which all the taxa respond to the same environmental gradient, where this gradient may result from the interplay of several biotic and abiotic factors that differ between sites (36). Coherence is positive when the taxa in the site-by-taxa matrix all respond to the same environmental gradient. Most extant well-sampled metacommunities display significant positive Coherence due to similarities in evolutionary history, ecological preferences or life-history trade-offs within communities (36). A significantly negatively Coherent site-by-taxa matrix reflects a high number of mutually exclusive taxa pairs creating checkerboard patterns (7,9,10). These checkerboard patterns do not have further underlying structure (in contrast to positive coherence patterns, Fig. 1), as there is no discernible gradient to which all the taxa respond. Negative co-occurrences and significant segregation/checkerboard patterns can be formed from strong competition, grazing/herbivory or strongly non-overlapping niches, all of which form similar metacommunity patterns due the presence of mutually exclusive pairs of taxa (29,31,36). A non-significant Coherence reflects no significant metacommunity structuring (Fig. 1). For metacommunities which have positive Coherence, the Turnover metric tests the amount of taxa replacement between sites (37). If taxa ranges are nested within each other, there is less Turnover than expected by chance along the gradient (significant negative). If there are more differences in site taxa composition along the gradient than expected by chance, Turnover is significantly positive and the structure is non-nested. Non-significant Turnover indicates a weaker structuring mechanism, termed a quasi-structure (Fig. 1) (30). The final metric, Boundary Clumping measures the extent to which taxa range limits cluster at the same sites across the environmental gradient (36). The range limits can be clumped (significant positive), hyperdispersed (significant negative) or random (non-significant).*

*Positive Coherence and negative Turnover result in nested metacommunities with taxa-poor sites being predictable sub-sets of taxa-rich sites, implying that species are dispersal limited (38). Nested metacommunities have been shown to be associated with a low degree of spatial connectivity and environmental variation (38) and have been shown to govern post-extinction dynamics (39).*

*Several taxa seemed to have unique taxa associations in each subdivision despite very similar community composition (Fig. S4. Tables S6-7), which naively we would expect to lead to the same taxa associations. So, what could underpin the 11/32 positive associations that differ between the middle shelf and deep subtidal environments? Variation in both inter-taxa interactions and environmental factors can cause organisms to exhibit behavioural plasticity, acting differently in different environments. Alternatively, differences in taxa associations for a given taxon may reflect the inclusion of several taxa with different environmental preferences and behaviors within one taxonomic group (e.g. Dickinsonia). However, as most Ediacaran taxonomic groupings are monotypic the differences in the behavior of the taxa associations in different environments are more likely due to plastic responses to, for example, variations in resource limitation or the presence of different competitors or ecosystem engineers.*

*Clumped species boundaries tend to be associated with the transitions between different biomes, where two biological communities mix, in contrast to hyper-dispersed species loss where species loss is evenly distributed across the range (30). Positive Coherence and Turnover with hyper-dispersed (negative) Boundary Clumping describes an Evenly Spaced metacommunity (Fig. 1). Where Coherence, Turnover and Boundary Clumping are all positive, the metacommunity is classed as Clementsian (Fig. 1), where groups of taxa with similar range boundaries co-occur and respond in a similar way to environment gradients (36,40). Taxa within Clementsian metacommunities respond synchronously to environmental gradients, suggesting physiological or evolutionary trade-offs associated with environmental thresholds (41), tend to result from high levels of environmental variability and spatial connectivity (38), and are found to be the most common (e.g. (42) ). When Coherence and Turnover are positive but there is no significant Boundary Clumping, the metacommunity is described as Gleasonian (Fig. 1) where each taxon reacts individualistically to environmental gradients (30).”*

8.      Lines 142-153: Similar patterns would be predicted by multiple ecological processes. For example, Connell's intermediate perturbation hypothesis led to similar predictions. Similarly, top-down control by predators would lead to lower diversity in environments with both low and very high abundance of predators. So, my question is: is there additional evidence that this is truly a consequence of ecological succession?

Predators have yet to evolve until the terminal Ediacaran, so they are not considered here. We have expanded on the links between this study and Connell’s theory on lines 251-255:

*This metacommunity structure and proposed succession is consistent with Connell’s disturbance theory (13) whereby intermediate stages of a community are most diverse because they enable both early and late colonizers to coexist (45). However, the lack of inter-specific competition found within small spatial scales of early-stage communities (11,12,14) suggests that interactions other than competitive exclusion were influencing community development.*

9.      Lines 165-167. I think I did not follow the reasoning here. If I got it then the approach used here implies that is impossible to record a positive association within this assemblage because the null hypothesis assumes 32/32 positive associations. Is that correct?

10.     Line 174-179: I did not follow this flow of ideas about ecological interactions.

There is some confusion here, and so we have clarified these points on Lines 306-317. We are referring not to the total number of possible associations (and how many are found), but given the number of associations found, how many are the same between different subsets.

*“Several taxa seemed to have unique taxa associations in each subdivision despite very similar community composition. (Fig. S4. Tables S6-7), which naively we would expect to lead to the same taxa associations. So, what could underpin the 11/32 positive associations that differ between the middle shelf and deep subtidal two environments? Variation in both inter-taxa interactions and environmental factors can cause organisms to exhibit behavioural plasticity, acting differently in different environments. Alternatively, differences in taxa associations for a given taxon may reflect the inclusion of several taxa with different environmental preferences and behaviors within one taxonomic group (e.g. Dickinsonia). However, as most Ediacaran taxonomic groupings are monotypic the differences in the behavior of the taxa associations in different environments are more likely due to plastic responses to, for example, variations in resource limitation or the presence of different competitors or ecosystem engineers.”*

11.     Line 240. There is something missing in this sentence, I think.

Our apologies, we have re-worded this section and removed the partial sentence.

12.     The discussion could be improved. There are entire paragraphs with no or just some introductory references, and I missed a truly synthesis with the literature of metacommunities, both from an evolutionary perspective (e.g., Thompson's Geographic Mosaic of Coevolution and Urban and Skelly 2006's evolving metacommunties) and from an ecological perspective (e.g., Guimaraes 2020's Annual review on ecological networks at different scales and Leibold's work on ecological processes in metacommunities)

We have substantially expanded on the introduction Lines 26-160 and so provide the necessary context there.

*“One of the most dramatic events in the history of Earth is the sudden appearance of animals in the fossil record during the Ediacaran period (635-541 Ma), after billions of years of microbial life (1–3). Ediacaran anatomies are particularly difficult to compare to modern phyla, which has hampered our understanding of Ediacaran evolution and how Ediacaran organisms relate to the Cambrian Explosion and extant animal phyla (4). Patterns of taxonomic, morphological and ecospace diversity change dramatically during the Ediacaran (5,6), which has led to the suggestion of several evolutionary radiations, corresponding to the Avalon, White Sea and Nama assemblages (1,7–9). These three assemblages consist of groupings of communities which occupy partially overlapping temporal intervals and water-depths, with no significant litho-taphonomic or biogeographic influence (7,8,10). The oldest assemblage, the Avalon (575-565 Ma), exhibits relatively limited ecological and morphological diversity (5,6), with only limited palaeoenvironmental influence on its composition and taxa interactions (11–14). The White Sea assemblage (558-550 Ma) shows a large increase in morphological diversity, including putative bilaterians (5), in tandem with a greater ecological diversity which includes the appearance of grazing, herbivory and wide-spread motility (15,16). These innovations are coupled to the development of dense communities with high community heterogeneity between environments (16,17) and increased taxa sensitivity to fine-scale environment (12,18). The Nama assemblage (549-543 Ma) includes the oldest biomineralizing taxa, and records a decrease in taxonomic diversity (5,19–21). The reduction in taxonomic diversity, “diversity drop”, and community composition complexity between the White Sea and Nama has been suggested to correspond to a post-White Sea extinction around 550 Ma, which eliminated the majority of Ediacaran soft-bodied organisms (9,22–24). This diversity drop has been suggested to be caused by either an environmental driven catastrophic environmental extinction or biotic replacement driven extinction (9,23,24,21). Recent work has shown that a biotic replacement driven extinction, whereby mobile metazoans out-competed soft-bodied Ediacaran organisms through bioturbation and ecosystem engineering, is unlikely due in part to prolonged co-occurrence of trace fossils with soft-bodied biota (1,25). Other currently unknown and/or unpreserved intrinsic causes behind a biotic replacement model cannot not be excluded at the moment.*

*Previous studies have focused primarily on defining the different assemblages and the underlying causes for the different assemblages (7,8,10), looking at taxonomic and morphological diversity between assemblages (5,26) with little investigation of how the ecological structure within the assemblages differs. The network structures of the co-occurrence of Ediacaran body-fossil and trace fossil taxa were compared by Muscente et al. (9) who found compartmentalisation of the assemblages within the total Ediacaran network. However, these prior cluster analyses of Refs (7,8,10) and network analyses of Ref 9 have not assessed the relative frequency of taxa co-occurrences within assemblages; i.e. whether they are were statistically different to what would be expected by random chance, nor compared the ecological structure within each assemblage to known ecological models.*

*In this study, we will investigate the structuring attributes within these assemblages using three analyses which have not been previous used to investigate Ediacaran macro-ecology. We used presence-absence data encompassing 86 Ediacaran localities and 124 taxa, with paleoenvironment, depth, lithology, time and assemblage data from (8,24) (Fig. S1). Ediacaran fossils are commonly found preserved in-situ, so their bedding-planes preserve near-compete censuses of the communities (15,27,27). This exceptional preservation means that ecological analyses normally reserved for modern communities can be applied e.g. (12,28).*

*First, we will use the “Elements of Metacommunity Structure” (EMS) framework to investigate emergent properties of groups of connected communities that may arise from taxa interactions, dispersal, environmental filtering and the interaction of these factors (29–31) (Fig. 1). Ediacaran communities vary in how much they are separated in time and space, from ecological to geological time-scales (8,9,13), and their organisms have been shown to have large dispersal ranges based on reproductive mode (28,32,33) and species occurrence over large space and time scales (34). Because the connectivity of these Ediacaran communities via dispersal has been established, here we define metacommunities as sets of fossil localities (communities) which are connected by the dispersal of many species (29). The EMS framework is a hierarchical analysis which identifies properties in site-by-taxa presence/absence matrices which are related to the underlying processes shaping taxa distributions (31), but to date has limited application to the fossil record (35). Three metacommunity metrics are calculated to determine the structure: Coherence, Turnover, and Boundary Clumping (29–31). The values and statistical significance of these metrics determine where the metacommunity fits within the 12 different metacommunity types within the EMS framework (Fig. 1), with different metric combinations indicating different underlying processes behind the metacommunity structure.*

*First, Coherence is a measure of the extent to which all the taxa respond to the same environmental gradient, where this gradient may result from the interplay of several biotic and abiotic factors that differ between sites (36). Coherence is positive when the taxa in the site-by-taxa matrix all respond to the same environmental gradient. Most extant well-sampled metacommunities display significant positive Coherence due to similarities in evolutionary history, ecological preferences or life-history trade-offs within communities (36). A significantly negatively Coherent site-by-taxa matrix reflects a high number of mutually exclusive taxa pairs creating checkerboard patterns (7,9,10). These checkerboard patterns do not have further underlying structure (in contrast to positive coherence patterns, Fig. 1), as there is no discernible gradient to which all the taxa respond. Negative co-occurrences and significant segregation/checkerboard patterns can be formed from strong competition, grazing/herbivory or strongly non-overlapping niches, all of which form similar metacommunity patterns due the presence of mutually exclusive pairs of taxa (29,31,36). A non-significant Coherence reflects no significant metacommunity structuring (Fig. 1). For metacommunities which have positive Coherence, the Turnover metric tests the amount of taxa replacement between sites (37). If taxa ranges are nested within each other, there is less Turnover than expected by chance along the gradient (significant negative). If there are more differences in site taxa composition along the gradient than expected by chance, Turnover is significantly positive and the structure is non-nested. Non-significant Turnover indicates a weaker structuring mechanism, termed a quasi-structure (Fig. 1) (30). The final metric, Boundary Clumping measures the extent to which taxa range limits cluster at the same sites across the environmental gradient (36). The range limits can be clumped (significant positive), hyperdispersed (significant negative) or random (non-significant).*

*Positive Coherence and negative Turnover result in nested metacommunities with taxa-poor sites being predictable sub-sets of taxa-rich sites, implying that species are dispersal limited (38). Nested metacommunities have been shown to be associated with a low degree of spatial connectivity and environmental variation (38) and have been shown to govern post-extinction dynamics (39). Several taxa seemed to have unique taxa associations in each subdivision despite very similar community composition. (Fig. S4. Tables S6-7), which naively we would expect to lead to the same taxa associations. So, what could underpin the 11/32 positive associations that differ between the middle shelf and deep subtidal two environments? Variation in both inter-taxa interactions and environmental factors can cause organisms to exhibit behavioural plasticity, acting differently in different environments. Alternatively, differences in taxa associations for a given taxon may reflect the inclusion of several taxa with different environmental preferences and behaviors within one taxonomic group (e.g. Dickinsonia). However, as most Ediacaran taxonomic groupings are monotypic the differences in the behavior of the taxa associations in different environments are more likely due to plastic responses to, for example, variations in resource limitation or the presence of different competitors or ecosystem engineers.*

*Clumped species boundaries tend to be associated with the transitions between different biomes, where two biological communities mix, in contrast to hyper-dispersed species loss where species loss is evenly distributed across the range (30). Positive Coherence and Turnover with hyper-dispersed (negative) Boundary Clumping describes an Evenly Spaced metacommunity (Fig. 1). Where Coherence, Turnover and Boundary Clumping are all positive, the metacommunity is classed as Clementsian (Fig. 1), where groups of taxa with similar range boundaries co-occur and respond in a similar way to environment gradients (36,40). Taxa within Clementsian metacommunities respond synchronously to environmental gradients, suggesting physiological or evolutionary trade-offs associated with environmental thresholds (41), tend to result from high levels of environmental variability and spatial connectivity (38), and are found to be the most common (e.g. (42) ). When Coherence and Turnover are positive but there is no significant Boundary Clumping, the metacommunity is described as Gleasonian (Fig. 1) where each taxon reacts individualistically to environmental gradients (30).*

*Secondly, to test whether within-assemblage community composition is correlated with depth, we use reciprocal averaging, a type of correspondence analysis which ordinates the sites based on their community composition (31), and then correlates the first-axis ranking (i.e. the order of sites based on community composition) with depth.*

*Thirdly, we will test to determine which pair-wise taxa co-occurrences are significantly non-random, and whether any non-random co-occurrences are positive or negative. We use a combinatorics approach to test whether species pairs are randomly distributed among sites (43). If co-occurrences are significantly non-random, this suggests a shared underlying ecological or evolutionary process. While the interpretation of co-occurrence data is complicated because co-occurrence does not necessarily correspond to interaction (44), here we interpret pair-wise interpretations to within the wider EMS framework where co-occurrences are not taken necessarily as direct taxa interactions, but could also indicate taxa environmental associations and/or disassociations.*

*Taken together these analyses enable us to test three hypothesises: We hypothesise that the increase in taxonomic and morphological diversity between the Avalon and White Sea assemblages is reflected in more ecological complexity in terms of increased taxa co-occurrences. We hypothesise that the total set of Ediacaran data exhibits strong metacommunity structure which reflects the previously recovered assemblages (7–9) and that the influence of environmental gradients increases between the Avalon and White Sea assemblages(18). Finally, we will use these analyses to test whether there is evidence of a catastrophic extinction event between the White Sea and Nama (9,22,45). Such an event would lead to negative Turnover, so a nested metacommunity structure (39).”*
