# Supplementary material for: Metacommunity analyses show an increase in ecological specialisation throughout the Ediacaran period
Source: PLoS Biol. 2022 May 17;20(5):e3001289. doi: 10.1371/journal.pbio.3001289 (PMC9113585; doi:10.1371/journal.pbio.3001289)
Supplement: Attachment — Submitted filename: review.docx [file pbio.3001289.s018.docx]

As a reviewer of the original submission, I am glad to have had the opportunity to provide a review of the revised manuscript of this paper. I thought the original paper had promise, but with numerous structural issues, which the other reviewers also recognized. I was pleased to see that they have attempted to address all the issues raised by the reviewers. The current manuscript is a marked improvement as a result.

That said, the current version still has numerous issues that will require at least one more round of revisions – some of these should have been caught by a careful re-reading prior to resubmission- so that is disappointing. I will try to enumerate these by line. I will also make a suggestion that I hope will improve the clarity of the paper.

1. Line 44. Is “the reduction in taxonomic diversity” different from “diversity drop”? Not clear!
2. Lines 73, 89. Both start with “First.” Reading further, the one on line 89 should be deleted.
3. Lines 73-87. Although there is evidence for larval transport and low provinciality, that does not indicate to me that these can all be treated as a metacommunity in the sense that ecologists would use it. Instead, acknowledge this but point out that for the purpose of this paper we can treat them as metacommunities.
4. Line 86-127.
   a. I went back to the original paper by Presley et al. (ref. 30) and found that there were actually 14 metacommunity types, if one includes Checkerboard and Random. Each of the six categories with positive coherence and significant positive or negative turnover has a non-significant, quasi- equivalent.
   b. Even with the revised figure caption and text, I was still confused about the relationships among the metrics and the community types. I was especially confused by what was meant by a “quasi-“ structure and what it implied about the metacommunity. Given that many of the analyses were consistent with a “Clementsian quasi-structure,” this needed further discussion.
   c. I have made a table (see below) because it clarified for me the properties of the various metacommunity types. I suggest using something like this in the next revision.
   d. I share with one of the other reviewers the issue of capitalization of the metrics. I advise not capitalizing them after they are introduced, and leave the capitalization to the metacommunity types, which are really what the paper should be about. As a comparison, “mean” and “standard deviation” are not capitalized.
   e. Lines 115-124 do not belong here! They are a fragment from the results (lines 304..) (a careful readover should have caught this – do not make this the reviewers job!)
5. Lines 139-142 and Figure 2. Not an accurate description of reciprocal averaging (correspondence analysis). The method ordinates the samples (sites) by their species composition (variable); it also ordinates the species by which site they are in. Variable ordination scores are averages of the case ordination scores and case ordination scores are averages of the variable ordination scores, ”thus “reciprocal averaging.” Both plot on the same axes. Depth is an independent variable, which can then be plotted against the RA axes to help with the interpretation. So, are the axes of Figure 2 are probably the scores for the sites and species on the first RA axis; the left-hand plot of depths is independent data, not used to do the ordination.

Also: make clear in caption to Figure 2 that this a reciprocal averaging ordination.

6. Line 172. Again, there are 14, not 12 models, all six illustrated models have quasi- equivalents
7. Lines 177-186. This paragraph badly needs rewriting.
   a. On line 178, you point to Figure 2, but then say nothing at all about it and then jump right away into Figure 3 and then back to Table 1. There needs to be a detailed explanations of both Figure 2 and Table 1, including what is meant by site scores.
   b. Looking at the results, it is clear that the relationship between depth and scores only holds true for the Nama, so that it will strongly influence the pooled results.
8. Lines 188-190. How does the low level of non-random associations compare to what is observed in modern communities or other paleontological examples? Is this unusually low or high? See: LYONS, S. K et al.. 2016. Holocene shifts in the assembly of plant and animal communities implicate human impacts. Nature, 529, 80-83.
9. Line 211-212. What is the simulated mean value of the metric? Not discussed in the text.
10. Lines 256-259. How are the succession stages determined? Needs more detail.
11. Lines 290, 334 – this is why there needs to be a more detailed explanation of quas-structures!
12. Line 334 – so this is the same structure as the White Sea?
13. Line 395-396. Is this a significant increase, given the large number of random associations? Again, we need context in terms of other communities.
14. Line 423-426. I am confused here. Based on table 1, the White Sea, except for the outer shelf, and the Nama have the same structure. The change is from the Avalon.
15. Line 468… this is Methods and should be so indicated.
16. Lines 469-487. This is confusing; both the RA and metacommunity analyses are being discussed in the same paragraph. Break these apart and make sure the RA is carefully described.

|  |  | **Metrics** | | |
| --- | --- | --- | --- | --- |
| **Community type** | **Properties** | **Coherence** | **Range Turnover** | **Boundary Clumping** |
| **Random** | No structure | Random |  |  |
| **Checkerboard** | High number mutually exclusive pairs; taxa do not respond to gradient | Negative |  |  |
| **Nested clumped** | Species poor communities subsets species rich communities; community synchronous response to gradient | Positive | Negative | Positive |
| **Nested random** | Species poor communities are random subsets species rich communities | Positive | Negative | Not significant |
| **Nested hyperdispersed** | Species poor communities subsets species rich communities; species respond individualistically to gradient | Positive | Negative | Negative |
| **Clemenstsian** | Community synchronous response to gradient | Positive | Positive | Positive |
| **Gleasonian** | Species respond individualistically to gradient | Positive | Positive | Not significant |
| **Evenly spaced** | Species spread along gradient with little overlap | Positive | Positive | Negative |
| **Quasi-nested clumped** | Species poor communities subsets species rich communities; community synchronous response to gradient, fewer turnover than random but not significant | Positive | Negative but not significant | Positive |
| **Quasi-nested hyperdispersed** | Species poor communities are random subsets species rich communities; fewer replacements than random but not significant | Positive | Negative but not significant | Not significant |
| **Quasi - nested random** | Species poor communities subsets species rich communities; species respond individualistically to gradient; fewer replacement than random but not significant | Positive | Negative but not significant | Negative |
| **Quasi-Clementsian** | Community synchronous response to gradient; more replacements than random but not significant | Positive | Positive but not significant | Positive |
| **Quasi- Gleasonian** | Species respond individualistically to gradient more replacements than random but not significant | Positive | Positive but not significant | Not significant |
| **Quasi-evenly spaced** | Species spread along gradient with little overlap; more replacements than random but not significant | Positive | Positive but not significant | Negative |
